# Layer-by-Layer Deposition of Low-Solid Nanochitin Emulgels Creates Porous Structures for High Cell Attachment and Proliferation

**DOI:** 10.1021/acsami.3c03421

**Published:** 2023-05-26

**Authors:** Ya Zhu, Esko Kankuri, Xue Zhang, Zhangmin Wan, Xin Wang, Siqi Huan, Long Bai, Shouxin Liu, Orlando J. Rojas

**Affiliations:** † Biobased Colloids and Materials Group, Department of Bioproducts and Biosystems, Aalto University, Vuorimiehentie 1, P.O. Box 16300, 02150 Espoo, Finland; ‡ Department of Pharmacology, Faculty of Medicine, University of Helsinki, Viikinkaari 5E, P.O. Box 56, 00014 Helsinki, Finland; § Bioproducts Institute, Department of Chemical & Biological Engineering, Department of Chemistry, and Department of Wood Science, The University of British Columbia, 2360 East Mall, Vancouver, British Columbia V6T 1Z3, Canada; ∥ Key Laboratory of Biobased Material Science and Technology (Ministry of Education), Northeast Forestry University, Harbin 150040, Heilongjiang, P. R. China

**Keywords:** 3D printing, Pickering emulsion, nanochitin, hierarchical porosity, cell proliferation

## Abstract

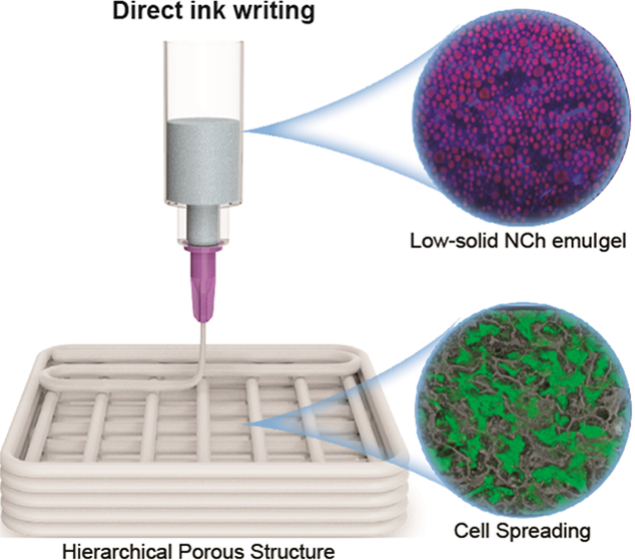

Direct ink writing (DIW) is a customizable platform to engineer complex constructs from biobased colloids. However, the latter usually display strong interactions with water and lack interparticle connectivity, limiting one-step processing into hierarchically porous structures. We overcome such challenges by using low-solid emulgel inks stabilized by chitin nanofibrils (nanochitin, NCh). By using complementary characterization platforms, we reveal NCh structuring into spatially controlled three-dimensional (3D) materials that generate multiscale porosities defined by emulsion droplet size, ice templating, and DIW infill density. The extrusion variables, key in the development of surface and mechanical features of printed architectures, are comprehensively analyzed by using molecular dynamics and other simulation approaches. The obtained scaffolds are shown for their hierarchical porous structures, high areal density, and surface stiffness, which lead to excellent modulation of cell adhesion, proliferation, and differentiation, as tested with mouse dermal fibroblast expressing green fluorescent proteins.

## Introduction

Lightweight, hierarchically porous constructs are suitable for adsorption, catalysis, and biotechnology platforms that take advantage of building blocks with a high surface-to-volume ratio.^1−3^ So far, synthetic precursors are preferred for creating porous structures through various routes, including replica, porogen-based and sacrificial templating.^4−6^ Such approaches take advantage of the possibility of adjusting initial synthesis conditions to control the characteristic pore size, fitting targeted applications.^7^ Naturally-derived colloids can be potentially used for the same purpose, since related properties are encoded in their structures. In this regard, a wide structural diversity can be added given the complex supramolecular assembly that emerges from colloidal interactions.^8,9^ Hence, multiscale porous structures have been achieved by introducing naturally harnessed materials;^10−12^ however, conversion into robust, hierarchical organizations has been challenging given the lack of control on multilevel assembly.

With the high freedom-of-structure design, 3D printing allows development of constructs with topological complexity and customizable shapes.^13,14^ Among various printing techniques, extrusion-based direct ink writing (DIW) distinguishes itself for the possibility to program layer-by-layer assemblies in confined spaces.^15,16^ DIW requires shear-thinning inks with efficient flow through nozzles; it also demands sufficiently high yield stress and storage modulus to ensure shape retention and distortion-free geometries.^17,18^ DIW has been used with a wide range of hydrogels to develop biobased materials.^19−21^ However, most DIW inks lead to 3D-printed constructs that lack porosity control, as a result of the effects of wetting, surface, and mechanical strength.^1,22,23^

The development of inks based on biogenic materials remains highly desirable to synthesize hierarchical porous constructs. To this end, emulsion-based inks have emerged as a suitable option for DIW due to the possibility to induce hierarchical pores,^24^ especially by controlling the state of the dispersed liquid phase.^18,25−27^ Compared with surfactant-stabilized emulsions, Pickering emulsions are especially suitable for DIW given that they lead to high colloidal stability during printing and subsequent drying.^28,29^ Notably, formation of pores with homogeneous size and distribution is possible with Pickering emulsions.^30,31^ The latter feature irreversible adsorption of particles, restricting droplet coalescence and deformation. Not surprisingly, processability, mechanical robustness, and fidelity have been reported with emulsion inks containing 58% solids that produced hierarchical porous ceramics.^26^ Our previous studies presented a universal methodology for generating low-solid (0.064 wt %) nanochitin (NCh)-stabilized Pickering emulsions with high internal phase volume, which were used to generate hierarchical porous structures via DIW.^29^ Unfortunately, the mechanical strength and fidelity of NCh-based structures were limited, given the low solid content and absence of interparticle adhesion and cross-linking. The latter issues can be addressed by considering the formulation and use of emulgels, i.e., multiphase systems that display a high elastic modulus or gel-like behavior.^32^ Such emulgels can be obtained at low solid concentrations by the effect of electrostatic interactions between oppositely charged groups that stabilize oil–water interfaces and gel the continuous phase, enabling excellent printability and fidelity after drying.^30^

**Figure 1:**
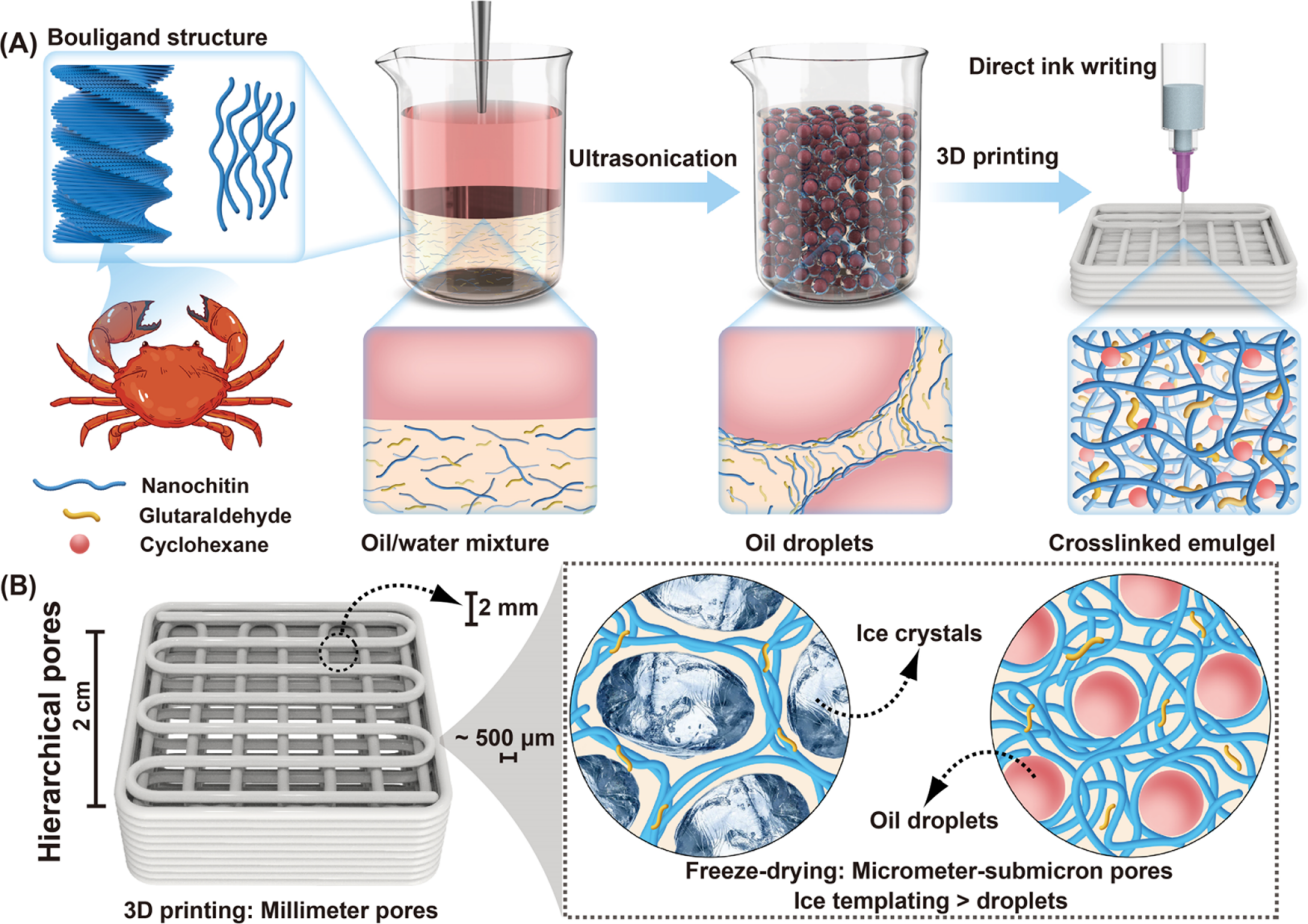
Schematic illustration of the development of hierarchical porous scaffolds. (A) Preparation of NCh-stabilized oil-in-water Pickering emulgels suitable for DIW. (B) Hierarchical 3D multiscale arrangement displaying three porosity levels: (1) millimeter-sized pores formed by designed DIW infill density; (2) micrometer-sized pores formed by ice crystal templating, and (3) submicron-sized pores formed by removal of the organic oil phase upon drying.

A turning point in the area requires solutions to the noted limitations, which is the focus of the recent study involving emulgel-based inks, namely, NCh-stabilized oil-in-water Pickering emulgels. Specifically, the power of our approach and the resulting materials are demonstrated by using emulsions with a continuous phase consisting of an aqueous suspension of NCh and a cross-linker (glutaraldehyde, Glu) and a dispersed volatile oil, cyclohexane (Figure 1A). We show that the NCh assembly forms interconnected networks at the surface and between the droplets (the organic phase). Emulgel inks subjected to shear forces and wall resistance, characteristics of extrusion through a nozzle, intensify the cross-linking of the continuous phase, ensuring desired printability. Emulgels (with solids content as low as 0.875 wt %) can then be easily transformed into lightweight, hierarchical porous scaffolds with suitable surface roughness and stiffness. In this study, we find that the microstructures formed are the result of conversion of the emulsion droplets into pores, leading to multiscale features under the additional effect of ice templating (Figure 1B). Together, the obtained hierarchical constructs are further shown as an effective platform for cell adhesion and growth in 3D cell culturing applications.

## Results and Discussion

### NCh-Based Pickering Emulgels for DIW

Chitin was partially deacylated to tailor the density of randomly distributed acetyl groups in the N-acetylglucosamine structure and to balance the interfacial energy.^33−35^ Hence, chitin nanofibrils (nanochitin, NCh) were used as Pickering stabilizers, and glutaraldehyde (Glu) was added to the aqueous phase to act as a cross-linker.^33^ NCh/Glu-stabilized cyclohexane-in-water Pickering emulgels were produced wherein oil droplets were jammed in the NCh/Glu network, preventing droplet transport/diffusion and deformation, as well as coarsening and coalescence (Figure 1A). Layer-by-layer deposition via DIW of the extruded emulgel led to 3D scaffolds of predesigned architectures. Multiscale porosity was developed at three distinctive dimensions (Figure 1B): (1) between 100 and 900 μm via the effect of the printed infill density; (2) ∼50 μm formed by ice crystal growth; and (3) ∼10 μm formed by templating Pickering emulgel droplets. Notably, the scaffolds did not suffer from the effects related to material composition to facilitate extrusion. Instead, our simple formulation enabled multi-material integration, as discussed in the next sections.

### Structure and Rheology of NCh-Based Pickering Emulgels

**Figure 2:**
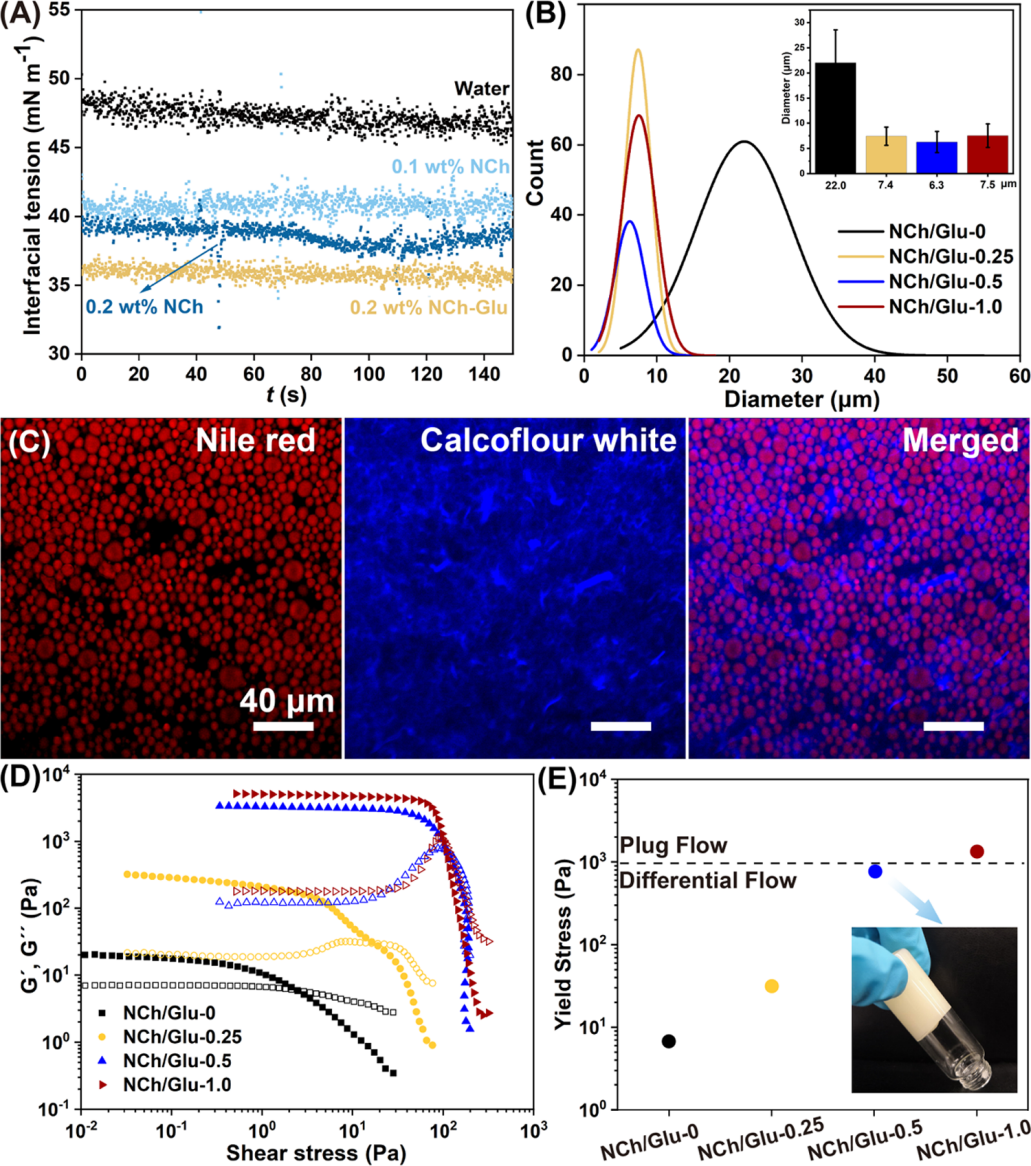
Structure and rheology of Pickering emulgels. (A) Time evolution of the interfacial tension between cyclohexane and water or aqueous suspension of 0.1 wt % NCh, 0.2 wt % NCh, and 0.2 wt % NCh-Glu-0.5. (B) Droplet size and distribution of emulgels stabilized with NCh/Glu-0, NCh/Glu-0.25, NCh/Glu-0.5, and NCh/Glu-1.0. The concentration of NCh in the aqueous phase was 0.6 wt % and the oil fraction was 50 v/v%. (C) Confocal images (multichannel) of emulgels stabilized with NCh/Glu-0.5. (D) Shear stress of emulgels at the given NCh/Glu ratio. The storage (*G*′) and loss (*G*″) moduli are indicated with filled and open symbols, respectively. (E) Yield stress of emulgels following the same color code used in (D). The dashed line denotes the transition from differential to plug flow. The photo inset displays the NCh/Glu-0.5 emulgel in an inverted vial (1.5 cm diameter).

A small amount of Glu was dispersed using tip sonication of the NCh aqueous suspension (0.6 wt %). The respective volumetric ratio corresponded to 10:0, 10:0.25, 10:0.5, and 10:1, leading to systems referred to as NCh/Glu-0, NCh/Glu-0.25, NCh/Glu-0.5, and NCh/Glu-1.0, respectively (Figure S1A). The given suspensions were colloidally stable, given the presence of protonated amino groups in NCh. The microstructures of NCh and NCh/Glu-0.5 comprised well-defined nanofibrils with few micrometers in length and 12–22 nm in width (Figure S1B,C). The typical indication of cross-linking was not apparent in AFM images obtained a short time after tip sonication. The behavior of NCh and NCh/Glu-0.5 at water–oil interfaces was examined by pendant drop tensiometry (Figures 2A and S2). Given the standard spherical shape of extruded droplets, we first optimized the concentration of NCh, up to 0.2 wt %. The interfacial tension between NCh-containing aqueous suspension and the organic phase was reduced with NCh loading and further decreased to 36 mN/m in systems containing the cross-linker, Glu. A lower energy input was required when premixing cyclohexane, NCh suspension, and Glu at such a stage, prior to emulsion preparation.

Our previous work demonstrated NCh-stabilized emulgels of high internal phase volume fractions. Herein, we used a 50% oil fraction to avoid collapse of the structure during cross-linking and drying. The stability of the Pickering system and microstructure of emulsions stabilized at different NCh/Glu ratios were assessed (Figure S3). All of the samples showed uniform droplet (organic phase) distribution, with average size decreasing from 22 to 7 μm with increased Glu addition (Figure 2B). Confocal images of the NCh/Glu-0.5 system formed with dyed NCh clearly indicated an interconnected blue contour around the oil droplets, a signature of NCh adsorption on the droplets (Figure 2C). The adsorbed and free NCh/Glu relaxed the typical need for high surface coverage, e.g., to prevent droplet coalescence. The same observation applied to emulsions solely stabilized with NCh (Figure S4). The NCh present in the continuous, aqueous phase underwent cross-linking under the condition of a limited free volume, given the high oil fraction. Such an effect arrested the oil droplets and prevented their diffusion. Finally, as shown in Figure S5, a new peak at 1703 cm^–1^ occurred in the NCh/Glu sample after cross-linking, indicating the presence of the C=N Schiff base following condensation reactions during cross-linking.^36^

Following the templating effect of ice crystals, lyophilization of the emulgel was applied to remove the liquid phase (oil droplets and water). As a result, only the fibril network remained (NCh or NCh/Glu; Figure S6). The obtained porous structures exhibited high mechanical integrity, with no collapse (Figure S6A). This is explained by the high interconnectivity of NCh/Glu upon dehydration and the formation of strong intra- and inter-hydrogen bonds. The presence of Glu in the continuous phase enabled cross-linking (Figure S6B), resulting in a dense and bulky structure that supported higher loads compared to those obtained from emulsions that were solely stabilized by NCh (Figure S7). Two levels of porosity were developed in the samples, with characteristic sizes of ca. 10 and 50–100 μm (Figure S6A,B). The smaller pores were generated by evaporation of the oil droplets (with pore size corresponding closely to the droplet diameter). Meanwhile, the larger pores were formed by NCh jamming upon ice crystal formation during freezing. Notably, the pores originated from droplets and ice crystals were retained after drying the systems that contained Glu (Figure S6B).

### NCh/Glu Ink Printability

Emulgels exhibited different flowability, as shown by inverted vials (Figure S8) and viscosity (Figure S9). NCh/Glu-0.25 demonstrated reduced flowability, while NCh/Glu-0.5 and NCh/Glu-1.0 did not flow. All three emulgels underwent pronounced shear thinning and displayed similar flow profiles.

The apparent viscosity decreased by several orders of magnitude by increasing the shear rate, from 10^–2^ to 10^2^ s^–1^ (values typically applied in DIW to ensure flow through the deposition nozzle, Figure S9). Remarkably, the deposited filaments displayed shape retention after printing. Oscillatory rheological measurements at low strain indicated that the storage modulus (*G*′) of the emulgel inks was dominant at low shear stresses. By contrast, the loss modulus (*G*″) of the emulgels was more relevant at high shear stresses, after crossing the yield stress (τ_y_, *G*′ = *G*″). Both *G*′ and *G*″ increased with the NCh/Glu ratio, indicating that cross-linking enhanced the elasticity of the NCh network, which was a result of highly connected, dense internal structures (nanofibrils and nanofibril–droplet complexes) surrounding the oil droplets. In practice, if the rheological properties are appropriate for DIW, a high τ_y_ and apparent emulgel viscosity are expected to generate a high printing pressure. This is the case if the maximum yield stress (τ_max_) within the nozzle is not sufficient to overcome τ_y_ of the emulgel. In such cases, plug flow occurs and prevents continuous printing. Thus, we calculated the radial τ within the deposition nozzle during printing (Figure 2E and discussion in the Supporting Information). The results indicate that the τ_y_ of NCh/Glu-1.0 was larger than the τ_max_ of the printing setup, which may block the needle. Meanwhile, NCh/Glu-0.5 met the printing requirements. A low Glu addition is desired given our targeted lightweight, highly porous, biocompatible scaffolds. Overall, considering the rheological behavior and emulgel structure, the NCh/Glu-0.5 system was chosen as the most suitable to fabricate emulgels for DIW.

### DIW Printing of Pickering Emulgels

Printing of the NCh/Glu-0.25 emulgel was not possible (Figure S11A), while different scaffolds were printed from NCh/Glu-0.5 emulgels, which formed stable and self-supporting structures (Figures S10, S11B, and S12 and Video S1). No evidence of collapse, deformation, shrinkage, or surface crumpling was observed in the NCh/Glu-0.5 system after freeze-drying (Figure S11C,D). Different designs were printed, showing the versatility of the emulgels to form complex and asymmetrical structures with high curvature. Furthermore, layers produced during printing were clearly identified before and after drying (Figure S12), indicating the ability of the emulgels to produce architectures with high geometrical complexity and fidelity (millimeter-scale resolution).

**Figure 3:**
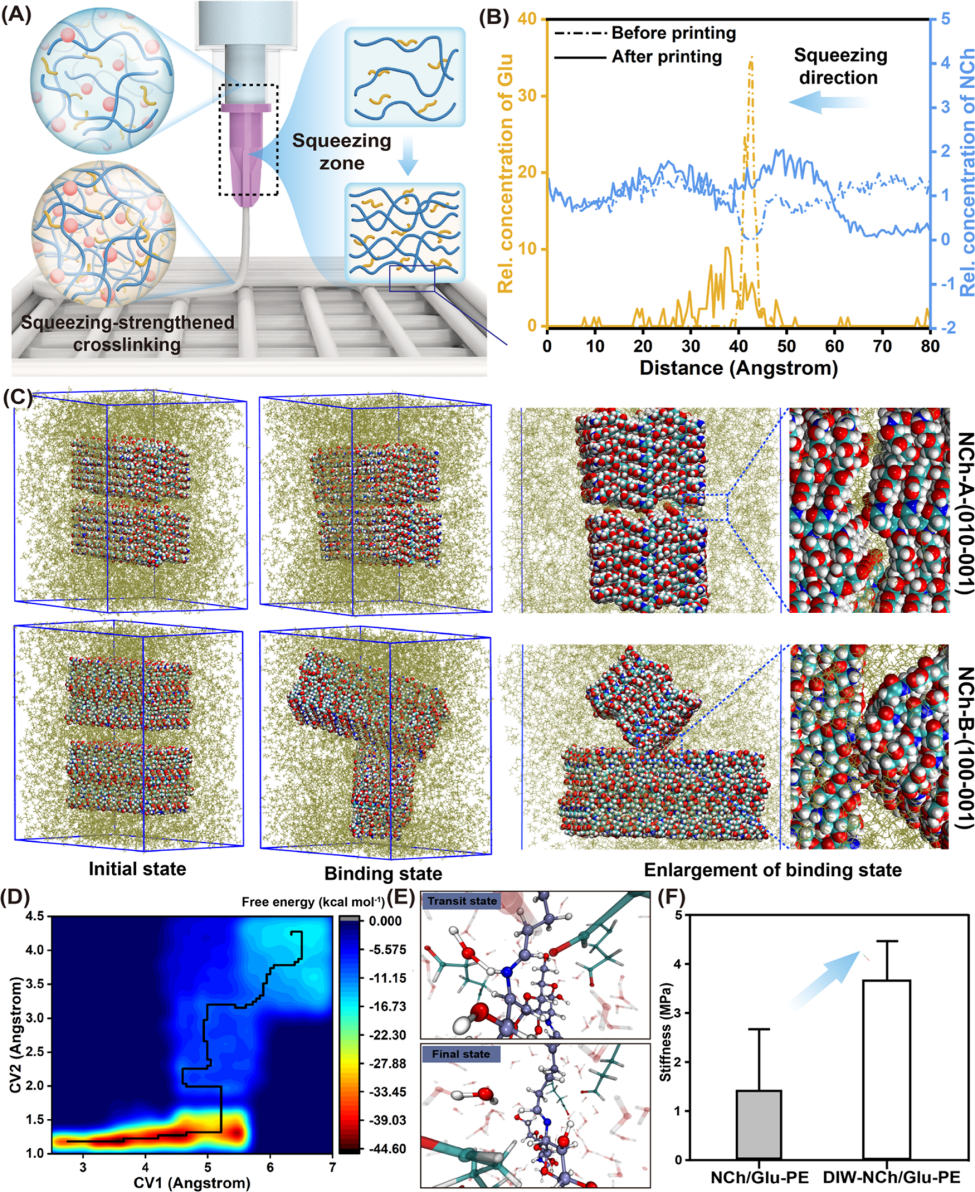
DIW printing of the emulgel and MD simulations. (A) Schematic illustration of the NCh, Glu, and oil droplet flow of Pickering emulgels in the printing head. (B) Relative distance change between NCh and Glu obtained from molecular dynamics (MD) simulations under a certain extrusion pressure. (C) Snapshots of classical MD (CMD) simulations of the interaction between chitin fibrils of different cross sections. The physical meaning of the parameters considers the hydrophilic (NCh-A-010-001, upper panel) or hydrophobic (NCh-B-100-001, bottom panel) surfaces. (D) Free surface energy obtained from ab initio MD (AIMD) simulations of NCh and Glu during cross-linking. (E) AIMD snapshots showing detailed cross-linking at different stages. (F) Stiffness of scaffolds produced from NCh/Glu Pickering emulgels (non-printed, NCh/Glu-PE) and those after the DIW process, indicated as DIW-NCh/Glu-PE, followed by immersion in a PBS solution.

Our next effort involved elucidation of the printing conditions to form Pickering emulgels suitable to produce filament grids, later tested as biomaterials. When initiating flow, the external as-set pressure was transferred to the emulgel to surpass its yield stress and wall resistance. The characteristics of the emulgels, such as the size of oil droplets and the network modulus of NCh/Glu fibrils, were influenced by dissipation processes; hence, the emulgels can be subjected to tighter packing to remove aqueous solutions in the interstitial spaces. This further deformed, squeezed, or even ruptured the emulgels before yield and flow. The process involved in the 3D printing of Pickering emulgels is illustrated in Figure 3A using a fixed volume of an emulgel. Under a given pressure during printing, an emulgel loaded in the printing syringe undergoes deformation. In turn, this influences the packing and cross-linking density (Figure 3A, left image). Because the oil droplets in the emulgel were not cross-linked, the pass-through modeling in the constriction zone was used to predict the activity of NCh and Glu molecules. In fact, the physical confinement in the nozzle caused NCh and Glu packaging, leading to a strengthened cross-linking density (Figure 3A, right image).

We carried out molecular dynamic (MD) simulations to rationalize the stated effects. First, we mimic the 3D printing process by adding an extrusion pressure in the simulation printing box and calculated the evolution of the relative concentration of NCh and Glu, depicted in the Z-coordinate (Figures 3B and S13). NCh moved over time according to the printing direction, from the top of the modeling box to the center. Simultaneously, Glu molecules diffused from a uniform distribution into the center of the simulation box, where they were concentrated. Thus, the distance between NCh and Glu decreased, leading to a higher contact probability, which implied that the relative density of NCh and Glu and cross-linking increased under confinement upon printing, as illustrated in Figure 3A.

To better understand the relationship between DIW and physicochemical characteristics of the associated scaffold, we further performed classical molecular dynamics (CMD) simulations to determine the fibril–fibril interfacial interactions and chemical cross-linking. Simulation details are provided in the Supporting Information (Figures 3C and S14). The first attempt involved two different models that were built to determine the interaction between NCh, NCh-A-(010-001), and NCh-B-(100-001) (see details in the Experimental Methods in the Supporting Information). In the simulation, surface–surface interactions occurred from single patch–patch interactions at a time, and a single NCh was initially placed within the vicinity of another NCh chain. The snapshots in Figure 3C show that neighboring chitin moved close and finally bonded tightly under the printing conditions simulated in the modeling box. The calculated data, including the center-of-mass (COM) distance (Figure S14A), interfacial non-bonded energy (van der Waals and electrostatic interactions, Figure S14B), and hydrogen bond number (Figure S14C), all together indicated strong NCh–NCh bonding. Thus, combining the simulation of CMD, an improved fibril density and interactions were observed in printed filaments compared to systems that were not subjected to extrusion.

The cross-linking pathway between NCh and Glu based on the reaction energy was calculated through ab initio molecular dynamics (AIMD) simulations. The AIMD trajectory in Figure 3D indicated that the cross-linking reaction occurred when the distances of C–N (CV1, in chitin) and H–O (CV2, in Glu) reached or were less than 5.2 and 1.3 Å, respectively. This provides insights into how the distance between the atoms affects the formation of Schiff base cross-linking, which in turn impacts the fibril–fibril density. Meanwhile, dynamic cross-linking snapshots (Figures 3E and S15) indicated individual atoms, formation of chemical bonds, and changes in the overall structure of the system. As shown in Figure 3E, from the transitional to the final state, a new Schiff base only formed when the two compositions (C and O in Glu and N and H in NCh) approached the limiting distance, which fits the free energy profiles (Figure 3D). This implies that a low activation energy was required for cross-linking to occur between NCh and Glu at a closer distance. Glu is a highly efficient cross-linker that leads to an increased network strength, further guaranteeing stability of the inner structure of the emulgel and its integrity during the printing process. Overall, the simulation results indicate that the DIW printing process leads to an increased fibril density and facilitates cross-linking between NCh and Glu.

In the following discussion, the NCh/Glu-based Pickering emulgels are referred to as NCh/Glu-PE. Meanwhile, the emulgels subjected to the DIW process are indicated as DIW-NCh/Glu-PE. The surface stiffness of the printed filaments (after freeze-drying and soaking into PBS solution) tested by AFM was higher than that of the non-printed scaffold (or precursor NCh/Glu-PE, Figure 3F), proving that DIW printing indeed enhanced the cross-linking strength of the filaments.

Figure S16 reveals the surface morphology of DIW-NCh/Glu-PE, which indicated large cracks, uneven mesopores, and dense nanopores on the surface. It is apparent that the wall resistance from the printing setup influenced the emulsion droplet packing density and caused dissipation, thus influencing the printing outcome. The inner structures of the filaments were highly interconnected and porous (Figure S16). The pore sizes of the samples are compared in Figure S17, showing that the NCh/Glu-PE sample had average pore sizes of 100 and 8 μm, respectively, while the DIW-NCh/Glu-PE sample showed average pore sizes of 50 and 7 μm, respectively (Figure S17B). During freezing and, due to different freezing temperatures, the initial oil droplets first solidified and then followed the formation of ice crystals in the NCh/Glu suspension. The small pores contributed to strong interactions between NCh and cyclohexane. The displacement of NCh occurred under the forces involved in the formation of ice crystals. Formation of large holes was further facilitated by high viscosity of the emulgel, which limited the growth of ice crystals. Herein, ice templating and oil droplets both played significant roles in defining the pore morphology. The latter can be tailored by controlling the physical interactions between ice and the substrate or by changes between the liquid and solid states. In sum, we have demonstrated that NCh/Glu-stabilized Pickering emulgels can be DIW printed into designed shape, enabling hierarchical, multilevel pores in solid architectures of ultralow solid content (0.875 wt %, Table S1), suitable for applications relating to surface attachment or fixation (high surface stiffness).

### Cell Culturing with Porous NCh Scaffolds

**Figure 4:**
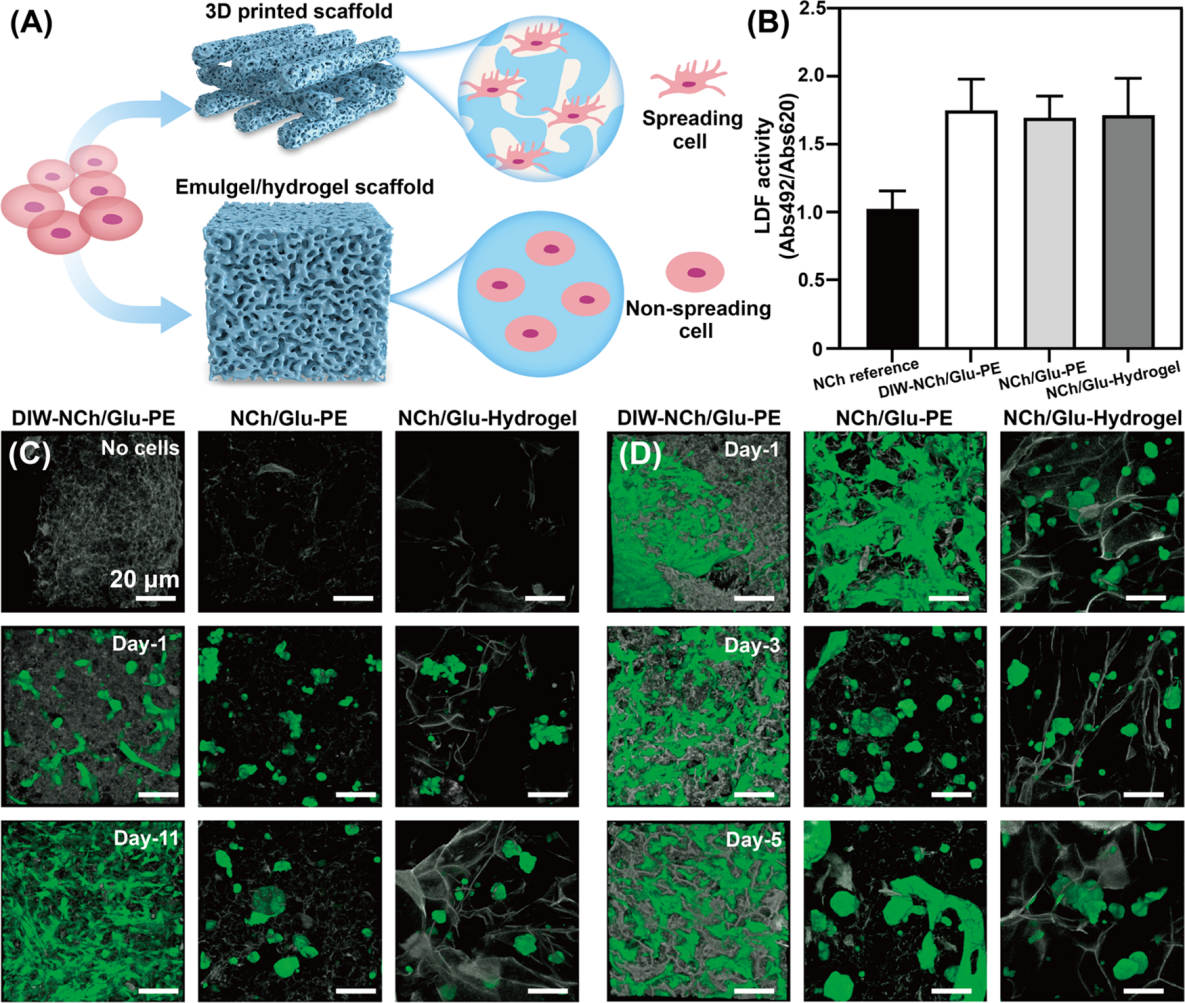
Cell culturing application of printed porous scaffolds. (A) Schematic showing cell growth and proliferation on DIW-NCh/Glu-PE and NCh/Glu-PE scaffolds. (B) Cell (LDF) activities. 3D confocal microscopy images of MDF-GFP cells living on the (C) non-cell medium-coated DIW-NCh/Glu-PE, NCh/Glu-PE matrix, and NCh/Glu-Hydrogel scaffolds and (D) cell medium-precoated scaffolds. The 3D confocal images were integrated from 30 z-stack images with a single stack thickness of 10 μm.

Taking advantage of the above findings, we next evaluated the compatibility of the hierarchical porous scaffolds in terms of cell–material interactions and their suitability for cell culturing. Compared to traditional hydrogel scaffolds, hierarchical porous scaffolds, as presented in Video S2, served as extracellular matrices for cells, providing a stiff microenvironment and a hierarchical porous structure that facilitated cellular mechano-sensation (Figure 4A).^37,38^ It is anticipated that the pore size and morphology of scaffolds can be tailored to drive a range of (biological) processes, including mass transfer of solutes, cellular interactions and organization, immune response, and drug delivery. The DIW-NCh/Glu-PE scaffolds were tested as biomaterials, which demand stringent tolerance to residues or impurities. A case in point is any excess or unreacted glutaraldehyde, which was effectively removed through washing. To test any related effect, the relative LDF cytotoxicity was evaluated by comparing the cellular responses to the matrices and by collecting the culture medium, e.g., to measure cell membrane leakage and cell death (see details in the Supporting Information). Mouse dermal fibroblast (MDF) expressing green fluorescent proteins (GFPs), MDF-GFPs, exhibited good viability after incubation in the DIW-NCh/Glu-PE scaffold and in the reference NCh/Glu-PE and NCh/Glu-Hydrogel (Figure 4B). Hence, any residual compounds had little or no biologically deleterious effects on the porous structure.

Subsequently, further studies focused on the capacity of the scaffolds to allow cell adhesion and proliferation, as well as cell–material interactions. The obtained scaffolds were immersed and washed with PBS before seeding them with an equal amount of MDF-GFPs. The microstructures of the DIW-NCh/Glu-PE scaffold, NCh/Glu-PE (see structures in Figure S6B), and NCh/Glu-Hydrogel (see structures in Figure S18) after soaking in PBS were compared (Figure 4C). The morphology of the DIW-NCh/Glu-PE scaffold exhibited a dense, multilayer, porous structure. By contrast, NCh/Glu-PE exhibited a loose, irregular, porous structure, while NCh/Glu-Hydrogel showed a non-uniform porous morphology with disconnected networks. The results indicate the high structural integrity of the scaffolds even after immersion in PBS solution. After 3-day incubation under regular cell culture conditions, MDF-GFPs were found by CLSM observation to be uniformly dispersed at low densities and on all three scaffolds. However, after 11 days, MDF-GFPs exhibited greater spreading and proliferation on DIW-NCh/Glu-PE compared to the other two scaffolds, demonstrating that only the printed scaffold provided significant support for cell growth and proliferation (Figure 4C). After evaluating the cellular compatibility with the scaffolds, to avoid any possible bad adaptability of cells to their new environment, we adopted a scaffold-protein-coating method with scaffold preincubation in the serum protein-containing culture medium of cells (Figure 4D). In comparison to the delayed adherence of the MDF-GFP cells to the crude material, the cells demonstrated prompt attachment already on the 1st day on DIW-NCh/Glu-PE and NCh/Glu-PE. However, the cells on NCh/Glu-PE exhibited decreased proliferation after 3 days, aggregated with non-adherent spherical shapes in the scaffold pores. After 5 days of incubation, cell adherence and spreading on the scaffold were well advanced on DIW-NCh/Glu-PE, whereas only a minor degree of increased cell spread from the aggregates in the scaffold pores was observed on NCh/Glu-PE. Video S3 indicates the cell activity in the printed scaffold. It appears that cells spread robustly and formed a cellular network on the DIW-NCh/Glu-PE scaffold, whereas cells were isolated into individual divisions or aggregates with phenotypes, showing less adherence and clustering and thus preferring adherence to each other rather than to the material.^39,40^

The most apparent factor explaining the excellent cell adherence to DIW-NCh/Glu-PE is its different surface stiffness and regularly porous morphology compared with those of other scaffolds tested. After extrusion via DIW, the obtained DIW-NCh/Glu-PE displayed high surface stiffness (Figure 3F) and extensional storage module (Figure S19), suitable as an environment for attachment and migration of MDF-GFPs. For comparison, the cells were difficult to spread on NCh/Glu-PE and NCh/Glu-Hydrogel (at the same solid content) owing to their irregularly porous structure and lower surface strength. Furthermore, the inner hierarchical porous structure with suitable pore size supported a favorable environment for diffusion of nutrients, allowing cell-to-cell communication through both paracrine and cell-contact-mediated signaling. The detailed molecular mechanisms behind the observed differential cellular behavior of MDF-GFP on the scaffolds require further investigation. In addition, enzymatic degradation of the scaffolds is an additional consideration. Control on the biodegradation by the lysozymes of partially deacetylated and glutaraldehyde-cross-linked chitin is the lead indicator.^41^ Overall, our results demonstrate that the DIW of NCh/Glu-based emulgels into hierarchically porous architectures leads to biocompatible scaffolds highly suitable for cell attachment, survival, and growth.

## Conclusions

In summary, we developed Pickering emulgels (inks) to assemble colloidal chitin nanofibrils into hierarchical porous architectures *via* layer-by-layer direct ink writing (DIW). Such approach allowed for an independent control of the microporosity, at the millimeter and micrometer scales, with ultralow NCh content (0.875 wt %). DIW printing of the emulgels generated high-fidelity, customizable scaffolds with high surface area and stiffness. Such scaffolds were shown as ideal platforms for attachment and proliferation of MDF-GFPs. Although we focused only on NCh, we foresee that the proposed Pickering emulgel strategy associated with DIW will open the possibility to synthesize complex hierarchical constructs from a wide variety of biobased colloids. Future work will broaden the applicability of emulgels, especially in the biomedical field, providing significant advances for tissue engineering.

## Experimental Section

### Materials

α-Chitin was obtained from fresh crabs (*Callinectes sapidus*) that were acquired in the local market (Helsinki harbor, Finland). All procedures including purification and deacetylation followed our earlier reported protocols.^34,38^ These steps yielded partially deacetylated chitin (DE-chitin, degree of deacetylation to be ∼27%). The obtained DE-chitin was redispersed into Milli-Q water (pH 3 with acetic acid) at 0.2 wt % solid content using a high-speed blender (T-25 Ultra-Turrax Digital Homogenizer, IKA, Germany) operated at room temperature for 5 min, which fully protonated the obtained amine groups. Afterward, microfluidization (M-110P, Microfluidics Inc., Newton, MA) was used to disintegrate the DE-chitin into NCh with a single pass at a pressure of 1200 bar. The obtained NCh was centrifuged at 11 000 rpm for 5 min to remove large particles, and the supernatant was collected, concentrated (0.6 wt %, pH 3.5), and stored at 4 °C for further use. The average aspect ratio of NCh was calculated to be ∼60 by counting the length and width from at least 100 nanofibers from Figure S1B. Glutaraldehyde (Glu) was purchased from Sigma-Aldrich (25% solution in H_2_O). Milli-Q water was obtained with a Millipore Synergy UV unit (18.2 MΩ·cm) and used throughout the experiments.

### Pickering Emulgels

We used an aqueous suspension (pH 3.5) of NCh/Glu (continuous phase) to prepare Pickering emulgels. Considering the high viscosity of the NCh suspension, Glu was dispersed with a titanium tip sonicator (Sonifier 450, Branson Ultrasonics Co., Danbury, CT) at a power level set at 10% strength with alternating on–off cycles (30 s, 5–2 s, respectively), which ensured homogeneous dispersion of Glu. The NCh suspension was mixed with Glu at volume ratios (mL/mL) of 10/0, 10/0.25, 10/0.5, and 10/1, coded as NCh/Glu-0, NCh/Glu-0.25, NCh/Glu-0.5, and NCh/Glu-1.0, respectively. Afterward, cyclohexane was emulsified with NCh/Glu at a 50/50 water-to-oil ratio by tip sonication under the same settings as detailed above. The temperature during sonication was controlled by using an ice/water bath. To avoid excess cross-linking and enable suitable printability, the freshly prepared emulgel was stored at room temperature for a maximum of 30 min (gelation time).

### DIW Printing

The emulgels were used as inks for DIW printing (BIO X, Bico Group CELLINK, Gothenburg, Sweden) using a pneumatic printing head. The device utilized a 3 mL pneumatic syringe provided by CELLINK and sterile blunt needles (plastic, Drifton A/S, Hvidovre, Denmark). Given designs were printed on plastic Petri dishes using rectilinear infill patterns and 20% infill density. Based on initial optimization, the moving speed of the printhead was 15 mm/s, the extrusion speed was 0.012 mm/s, and the extrusion pressure was controlled in the range of 20–40 kPa. After printing, the scaffolds were frozen overnight in a refrigerator (−18 °C) followed by lyophilization for 12 h (Free Zone 2.5, Labconco, MO, USA), wherein the water and oil in the scaffolds were removed. Then, any remaining, unreacted Glu was removed by washing the dried scaffolds multiple times with Milli-Q water. Simulations of the printing and its effect on the filaments were performed as described in the Experimental Methods in the Supporting Information.

### Characterization

The interfacial tension of the suspensions was measured using an optical tensiometer (Theta Flex, Biolin Scientific Oy, Finland). Briefly, Milli-Q water, NCh, and NCh/Glu suspension (pH = 3) were loaded into the tip, and cyclohexane was loaded into a quartz cuvette. The interfacial tension tests were performed optically using pendant drop shape analysis. The shape of the drop hanging from a needle was determined from the balance of forces, which included the surface tension of the liquid being investigated. The surface or interfacial tension was related to the drop shape according to
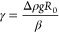
where γ is the surface tension, Δρ is the density difference between fluids, g is the gravitational constant, *R*_0_ is the drop radius of curvature at the apex, and β is the shape factor. β was defined through the Young–Laplace equation with computational methods using iterative approximations.

The morphology of Pickering emulgels was observed using an optical microscope (BX53M, Olympus Corp., Tokyo, Japan) with a 20× objective lens. A drop of the given emulsion was placed onto a microscope slide and covered with a glass coverslip (Assistent, Sondheim, Germany). A confocal laser scanning microscope (CLSM) with a 20× objective lens (DMRXE, Leica, Germany) was used to observe the microstructure of the emulgels. Cyclohexane was stained with Nile red solution (1 mg/mL in ethanol) at a ratio of 1/25 overnight. NCh/Glu suspensions were stained by Calcofluor white mixed with magnetic stirring for 1 h. The emulsions stabilized by NCh/Glu were stored for 1 h prior to observation. A drop (6 μL) of the dyed samples was placed on a microscope slide and covered with a glass coverslip. The coverslip was quickly fixed by nail polish to avoid oil evaporation. The excitation/emission spectrum for Nile red and Calcofluor white stain were 488/539 and 365/435 nm, respectively. Merged fluorescent images were processed by Photoshop software.

The rheological behavior of the emulgels was measured with a rheometer (MCR 302, Anton Paar, Germany) equipped with a parallel plate (PP25) and a gap fixed at 1 mm. All samples were presheared at a shear rate of 10 s^–1^. The shear viscosity was monitored at varying shear rates (10^–2^–10^2^ s^–1^). For dynamic viscoelastic measurements, the linear viscoelastic range was determined with a strain sweep (0.01–100%) at a fixed frequency of 10 rad/s. To determine the yield stress of the materials, oscillatory measurements were carried out at a constant frequency of 1 Hz and increasing stress from 10^–2^ to 10^3^ Pa using a rheometer (MCR 302) equipped with a parallel plate (PP25) and a gap fixed at 1 mm. All samples were presheared at a shear rate of 10 s^–1^. All measurements were performed at 25 °C. The extensional storage modules of the scaffolds were measured by using a rheometer (MCR 702, Anton Paar, Germany) using samples of the same shape (10 m × 10 cm × 5 mm) that were kept soaked in phosphate-buffered saline during the test.

The microstructure of the freeze-dried scaffold was observed by a scanning electron microscope (SEM, Zeiss Sigma VP, Carl Zeiss AG, Oberkochen, Germany) operated under vacuum and at an accelerated voltage of 2.5 kV. The cross-sectional structure was revealed by clean knife cuts. The samples were sputter-coated with platinum before imaging. The stiffness (Young’s moduli) of Pickering emulgels before and after DIW printing (freeze-drying then soaking with phosphate-buffered saline) was measured by atomic force microscopy (AFM, JPK-Bruker NanoWizard IV XP, Germany) with an RTESPA-525 probe (*k*_c_: 200 N/m, *f*_0_: 525 kHz).

### Cell Seeding of Printed Scaffolds

Primary mouse dermal fibroblasts (MDFs) were isolated from skin samples of adult mice and transduced with the GFP-expression lentivirus (pLV-PGK/GFP) as described previously.^42,43^ A 100% transduction efficacy was ascertained at each passaging and before experimentation by evaluating with a fluorescence microscope with 10× and 20× objective lenses (EVOS, Thermo Fisher Scientific Inc.). The GFP-expressing cells (MDF-GFP) were cultured in complete culture medium (Dulbecco’s modified Eagle medium, DMEM, Gibco 22320022, Thermo Fisher Scientific Inc.) supplemented with 10% heat-inactivated fetal bovine serum (Gibco 10500-064), 2 mM l-glutamine (Gibco A2916801), and antibiotics (Gibco 15140-122, penicillin G 100 U/mL, streptomycin 100 μg/mL and Gibco 15290-026, amphotericin B 250 ng/mL) in a humidified atmosphere at +37 °C supplemented with 5% CO_2_. The medium was changed twice a week, and the cells were passaged at sub-confluency. One day before experimentation, the matrices were incubated in either phosphate-buffered saline (uncoated) or in complete culture medium (coated). 3 mm × 3 mm pieces of matrices were cut with a surgical scalpel and placed separately in 3.5 cm diameter culture dishes. The cells were counted and viability was assessed using a Countess Automated Cell Counter (Thermo Fisher Scientific Inc.), wherein the cells constantly maintained a 98% viability at passaging. MDF-GFP cells (10 million viable cells/mL) were carefully pipetted onto each matrix (4 × 10 μL) and incubated for 20 min at cell culture conditions. Thereafter, a second addition of cells (4 × 10 μL) to each matrix was applied and the matrices were incubated for 45 min at cell culture conditions. 1.5 mL of complete culture medium was added to each dish. Matrices and cells were incubated for indicated time periods and were only removed from the cell culture incubator before imaging.

For imaging at each indicated time point, matrices were gently lifted using cell lifters (08100240, Fisherbrand Cell Lifter, Thermo Fisher Scientific Inc.) and flipped upside down to 35 mm glass-bottom dishes (P35G-1.5-14-C, MatTek Corporation) to allow reflective confocal imaging from below. Fresh medium was added to each dish, which was kept at a humidified +37 °C atmosphere during imaging. Before imaging, the excess culture medium was gently removed to fix the sample on the glass surface. Imaging was performed with a Leica TCS SP8 CARS confocal with a DMI8 microscope using the HCX IRAPO L 25x/0.95 water-immersion objective (Leica, Germany) and Leica Application Suite X (LAS X 3.5.2, Leica). Dual channel Z-stack images were obtained at 405/488 nm excitation/emission windows (410–470 and 500–550 nm, respectively). Z-stack images of 1024 × 1024 pixels were obtained using 1 μm Z-step size and 600 Hz imaging speed with a pixel size of 455 nm. The obtained images were imported into ImageJ software (Fiji) for further analysis.

## Supporting Information Available

Interfacial tension of NCh and NCh/Glu; optical microscopy images of emulgels stabilized by NCh/Glu-0, -0.25, -0.5, and -1.0; confocal images of Pickering emulgels stabilized by 0.6 wt % NCh; FTIR of NCh and NCh/Glu-0.5; SEM of freeze-dried emulgels stabilized with NCh/Glu-0 and -0.5; compression curves of freeze-dried scaffolds; visual appearance of Pickering emulgels at different NCh/Glu ratios stored in the inverted containers; scaffolds printed with NCh/Glu-0.25 and -0.5 emulgels; snapshots of molecular dynamics simulations of relative distance between NCh and Glu molecules; classical molecular dynamics simulations for the assembly behavior of NCh, including the distance of the center of mass among NChs, non-bonded interactions, and hydrogen bonds among NChs; snapshots of the ab initio molecular dynamics simulation of the cross-linking reaction process between NCh with Glu molecules; SEM images of the porous structures of the printed scaffold and NCh/Glu-hydrogel after freeze-drying; and composition of Pickering emulgels used for DIW printing (PDF)
Video S1: printability of the NCh/Glu-0.5 Pickering emulgel (MP4)
Video S2: 3D CLSM video of the DIW-NCh/Glu-PE scaffold in phosphate-buffered saline (AVI)
Video S3: 3D CLSM video of MDF-GFP cells on DIW-NCh/Glu-PE scaffolds (cell medium-coated) for 1 day (AVI)

## Supplementary Material

am3c03421_si_002.mp4

am3c03421_si_003.avi

am3c03421_si_004.avi
