# Supplementary material for: Layer-by-Layer Deposition of Low-Solid Nanochitin Emulgels Creates Porous Structures for High Cell Attachment and Proliferation
Source: ACS Appl Mater Interfaces. 2023 May 26;15(22):27316–26. doi: 10.1021/acsami.3c03421 (PMC10251351; doi:10.1021/acsami.3c03421)
Supplement: Supplementary file 1 — am3c03421_si_001.pdf [file am3c03421_si_001.pdf]

Supporting Information

Layer-by-Layer Deposition of Low-Solids Nanochitin Emulgels Create Porous Structures for High Cell Attachment and Proliferation

Ya Zhu,[1] Esko Kankuri,[2] Xue Zhang,[1] Zhangmin Wan,[3] Xin Wang,[4] Siqi Huan,[4] Long Bai,[3,4] Shouxin Liu,[4*] Orlando J. Rojas[1,3*]*

[1] Biobased Colloids and Materials group, Department of Bioproducts and Biosystems, Aalto University, Vuorimiehentie 1, P.O. Box 16300, 02150 Espoo, Finland

[2] Country Faculty of Medicine, Department of Pharmacology, University of Helsinki, Viikinkaari 5 E, P.O. Box 56, 00014 Helsinki, Finland

[3] Bioproducts Institute, Department of Chemical & Biological Engineering, Department of Chemistry, and Department of Wood Science, 2360 East Mall, The University of British Columbia, Vancouver, BC V6T 1Z3, Canada

[4] Key Laboratory of Biobased Material Science and Technology (Ministry of Education), Northeast Forestry University, Harbin 150040, P. R. China

***Corresponding authors:**

E-mails: orlando.rojas@ubc.ca; long.bai@nefu.edu.cn; liushouxin@nefu.edu.cn

This Supporting Information document contains nineteen (19) figures and one (1) table in nineteen (19) pages. Three videos are supplied as separate files.

Experimental methods:

Atomic force microscopy (AFM). A spin coater was used to prepare ultrathin films for observation in AFM. Briefly, the NCh suspension was diluted with pH 3 Milli-Q water to 0.01 wt%, a silica plate was cut into 1 cm^2 slice and was treated with UV light for 15min. A 10 μL aliquot of the suspension was then placed on the silicon surface and ran with 3000 rpm to spinning. The morphology of NCh and NCh/Glu were observed by using a Multi Mode 8 Atomic Force Microscopy (AFM) equipped with a NanoScope V controller (Bruker Corporation, Billerica, MA, USA). Tapping mode in air using NCHV-A probes (Bruker) with a tip radius around 10 nm was applied.

Infrared spectroscopy (IR). The IR measurements were performed on a PerkinElmer FT-IR Spectrometer Frontier (Waltham, Massachusetts, USA). Spectra were recorded from 4000 to 650 $cm^{-1}$with a resolution of 4 cm^{-1}. **Figure S5** demonstrates that the crosslinking of NCh and Glu followed Schiff-Base condensation and crosslinking because of the development of imine bond (N=C, 1700 cm^{-1}) in the sample.[1]

Rheological behavior. For the calculation of radial τ within the deposition during printing, below equation is used:[2]

$$\tau = \frac{\Delta P}{2L} r$$

where ΔP is the maximum pressure applied at the nozzle, r is the radial position from the center of the nozzle, and L is the nozzle length. Using ΔP = 4 × 10^4 Pa, r = 6.3 × 10^{-4} m, and L = 1.27 × 10^{-2} m, the calculated τ_{max} for the nozzle is approximately 1.0 × 10^3 Pa. The τ_y for NCh/Glu, NCh/Glu-0.25, NCh/Glu-0.5, and NCh/Glu-1.0 was 6.6, 31.3, 821, and 1298 Pa, respectively. In our study, the printing pressure was set between 20 to 40 kPa. The reason for such low printing pressure was that the solid content of the developed NCh/Glu-stabilized Pickering emulsion was quite low and the droplets within the emulgels were deformable. The droplets

may undergo irreversible deformation and eventually break up under a high printing pressure, which prevents pore generate at submicron levels.

Mechanical properties. NCh/Glu-PE and DIW-NCh/Glu-PE were prepared with 50% oil volume Pickering emulgel. The NCh/Glu-Hydrogel was prepared from a 0.3 wt% NCh suspension. Three samples were shaped using the given plastic mold. The compression test was performed to evaluate the mechanical performance by employing Dynamic Mechanical Analysis (DMA Q800, TA, USA) operated at room temperature. In the compression test, samples were equilibrated with DI water. Then, the wet samples were placed in the DMA for tests in compression mode at a maximum compression strain of 70% (at a strain rate of 100 μm/s).

Molecular dynamic (MD) simulation. MD simulation was utilized to gain insight into the chitin and glutaric dialdehyde interactions under external pressure. The polymerization degree of chitin was assumed as 160.[3] Glutaraldehyde (8.5) molecules were distributed between two chitin molecules in the middle layer, as show in **Figure S13**, as well as 4000 water molecules with chitin and glutaraldehyde. We applied periodic boundary conditions in all directions, and the size of the resulting simulation box was 6 nm× 6 nm× 8.5 nm. The densities of chitin and glutaraldehyde molecules placed in the simulation box were calculated to be 0.476 and 0.025 g/cm^3, respectively. Atoms in the bottom layer with 3Å in thickness were restrained, and atoms in the upper layer with 3Å were loaded, at a pressure of 20 kcal/mol/Å, which was estimated from experiments. Before starting the simulation, geometric optimization was carried out for energy minimization. The NVT ensemble was used with constant temperature maintained at 298 K by the velocity rescale thermostat. In the NVT simulation, time scale was set as 0.5 fs and simulation steps was 200000, for a simulation time of 1 ns. The Ewald method was applied to calculate the Coulomb interaction. The atom-based method was chosen to calculate the van der Waals interaction. All the calculations in this work were carried out using a Forcite package

in Material Studio. The COMPASSII force field was adopted for the simulation, which is widely applied to organic/inorganic molecules and calculating the properties of materials.[4]

Classical molecular dynamic (CMD) simulation. The α-chitin crystal was built according to the crystallographic vector reported in previous literature,[5] using the BBBB configuration. The α-chitin crystal was assumed with a size of 8.264 nm for 001 crystallographic plane, 3.778 nm for 010 crystallographic plane, and 2.8494 nm for 100 crystallographic plane. To mimic our experimental process, the aminoacetaylated groups on the 001-110 plane were assumed as amino groups (NH_2). The α-chitin crystal with BBBB configuration was built by using our own awk script. In addition, glutaraldehyde molecules were built by CHARMM-GUI ligand module.[6] The complex configuration was established by using PACKMOL with a box dimension of 12 nm×9nm×13 nm.[7]

All the CMD simulations described herein were conducted by using Gromacs,[8] version 2020.7, with the CHARMM36 Force Field for carboxylate[9] and TIP3P water model.[10] All the topology files were made by using CHARMM-GUI.[11] Firstly, the complex structure underwent energy minimization by using both steep descents and followed conjugated gradient methods, with the maximum force of 50 kJ/mol/nm. The first equilibrium simulation was carried out in NPT ensemble at 303.15 K and 1 bar ambient pressure for 1 ns simulation to equilibrate the water and glutaraldehyde molecules. Then, the production simulation trajectories were propagated in NPT ensemble for 100 ns. During all the simulations, the particle mesh Ewald (PME) approach was used to evaluate long-range electrostatic interactions.[12] The short-range interactions, including van der Waals interaction as well as short-range Coulombic interactions, used a cut off at 1.2 nm. The visualization in this study was done by VMD package, version 1.9.3.[13] The energy analysis was done by gmx energy utility. The distance of mass center and hydrogen bonds between two alpha-chitin crystal was done by Tcl script. The criteria for hydrogen bonds included 0.35 nm and 30°.

***Ab initio* molecular dynamic (AIMD) simulation.** A simplified model was utilized To simulate chitin nanofibers crosslinked with glutaraldehyde molecules, where N-acetylglucosamine and glucosamine units with β-1,4 linkage were used and built with the Avogadro package.[14] Then, water and glutaraldehyde molecules and N-acetylglucosamine-β-1,4-glucosamine structures were assembled in a orthorhombic periodic box with dimensions of 15.0 Å × 20.0 Å × 15.0 Å. *Ab initio* molecular dynamics simulations (AIMD) were carried out by using the QUICKSTEP module of the CP2K package (Version 9.1).[15] Before AIMD, the structural optimization was used to eliminate the unreasonable contact between each atom. Atomic forces were estimated using density functional theory (DFT) calculations with the Perdew-Burke-Ernzerhof (PBE) exchange-correlation functional with D3 (BJ) dispersion correction.[16,17] The DZVP-MOLOPT-SR-GTH basis set was employed for all atoms.[18] The total AIMD simulation is 350 ps with a time step of 1 fs. The temperature of the simulation was maintained at 298.15 K using the Canonical Sampling through Velocity Rescaling (CSVR) thermostat coupled to the system with a time constant of 200 fs.

To obtain the free energy profile of the reaction path, meta-dynamics simulation was carried out.[19] The optimal reaction path of free energy was searched by MULE package. [20] Herein, the collective variables 1 (CV1) were defined as the distance between C (from aldehyde of glutaraldehyde molecule) and N (from amino of glucosamine unit). While the CV2 was defined as the distance between O (from aldehyde of glutaraldehyde molecule) and two hydrogens (from amino of glucosamine unit). Repulsive Gaussian-shaped potential hills with a height of 0.5 kcal/mol and a width of 0.1 were added to the potential every 5 steps for all collective variables. The coordination for x and y axis is 6.3 and 4.2 angstrom corresponding to the initial configuration. Free energy decreased to 30 kcal/mol when the vicinity C-N (CV1) and H-O (CV2) reach the distance to 5.2 and 1.3 angstrom, respectively. As the breakage of aldehyde bond by amino group attack, H and O closed to each other from 2.0 to 1.3 angstrom, hence a H_2O molecular produced with the reaction reach to a relative steady state. Afterward,

the bond distance between C and N decreased continuously, owing to the C-N bond transferred into C=N bond. Since this Schiff base reaction is reversible and the H_2O molecular obtained from last step remains exists in the system, the free energy showing slightly increase at the final state from 38 to 28 kcal/mol.

LDH cytotoxicity. The cell-matrix incubation medium was collected after a 5-day-incubation and the lactate dehydrogenase (LDH) activity was measured using the colorimetric LDH cytotoxicity detection kit plus (Roche 04744926001, Millipore Sigma Burlington, MA, USA) according to the manufacturer's instructions and as described earlier. [21] Briefly, 100 μL of the assay reagent was mixed with 100 μL of the cell culture sample supernatant. After a 30-min incubation in the dark, the reaction was stopped, and spectrophotometric analysis was performed using a microplate reader. Optical density results at 492 nm were corrected with those at 620 nm. Levels of basal LDH activity measured from naive culture medium samples were subtracted from the obtained values before analysis.

Supporting figures:

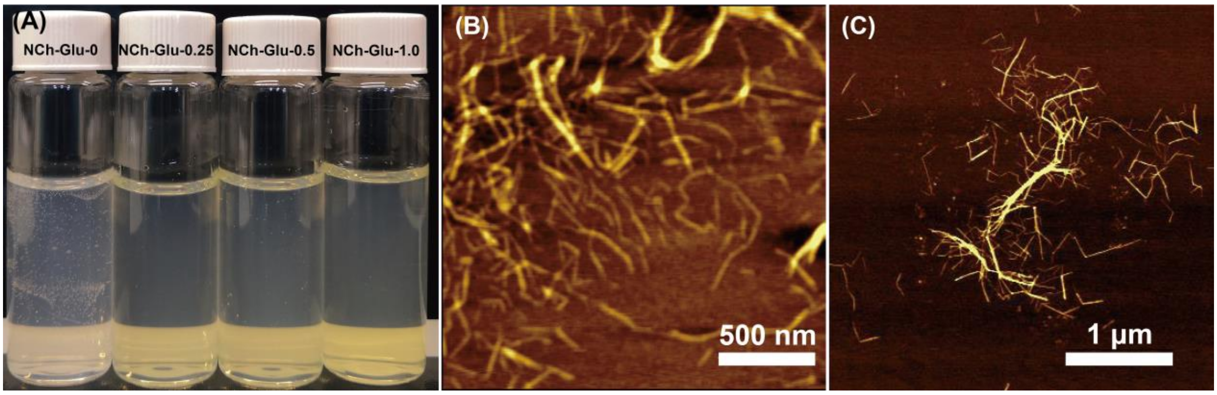

Figure S1. (A) Visual appearance of 0.6 wt% NCh suspension with different Glu addition. Atomic force microscopy (AFM) images of (B) NCh/Glu-0 and (C) NCh/Glu-0.5, respectively.

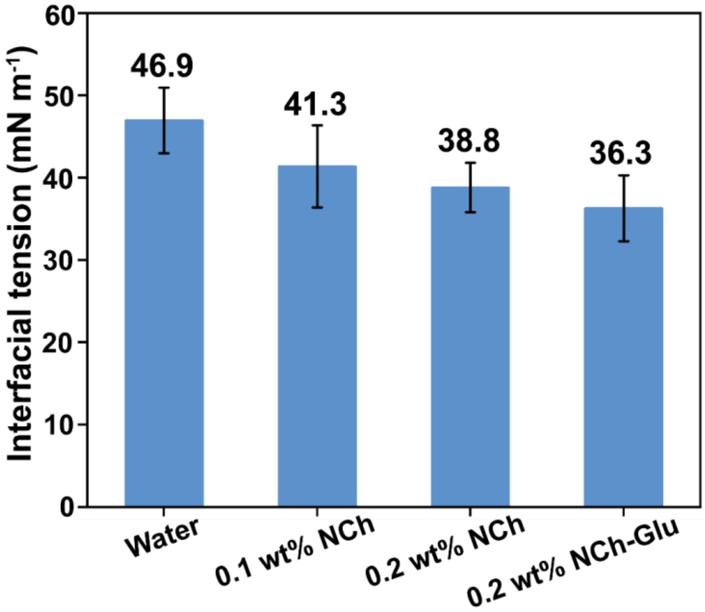

Figure S2. O/W interfacial tension corresponding to NCh and NCh/Glu.

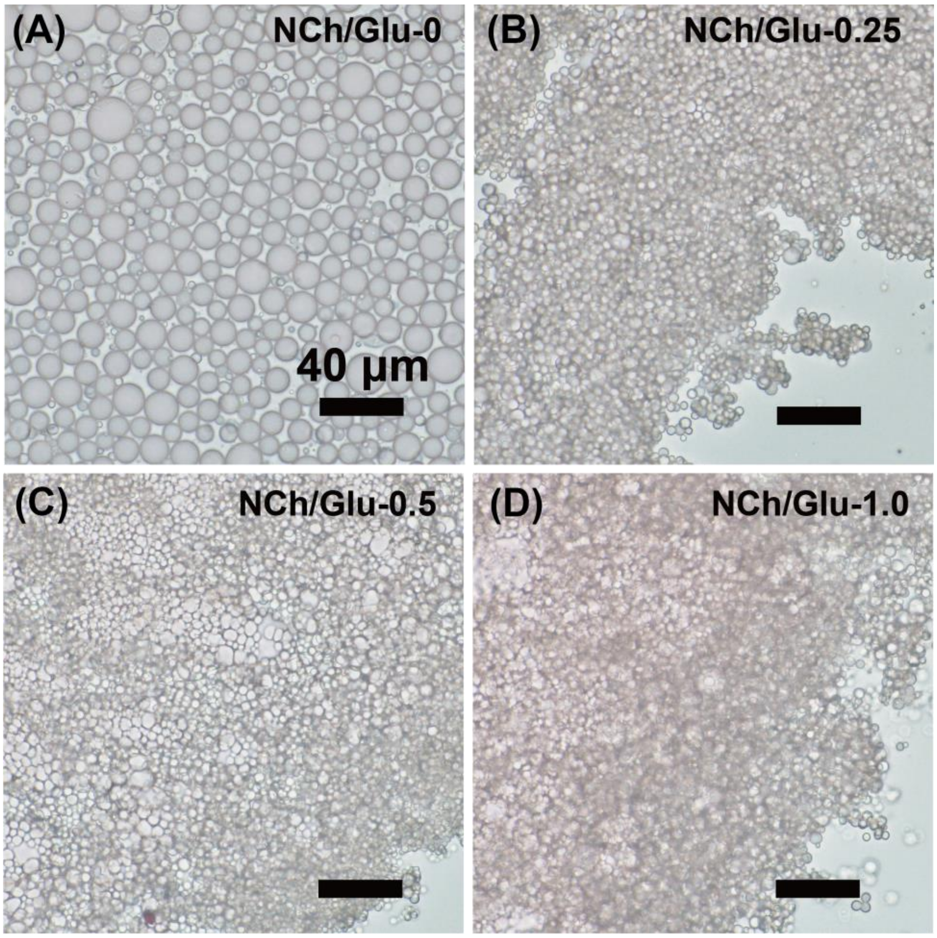

Figure S3. Optical microscopy images of emulgels stabilized by (A) NCh/Glu-0, (B) NCh/Glu-0.25, (C) NCh/Glu-0.5, and (D) NCh/Glu-1.0. The oil volume is 50%. The concentration of NCh in continuous phase is 0.6 wt%. Note the individual droplets for Glu-crosslinked samples at the edge of the images, which are otherwise difficult to visualize in the image due to clustering.

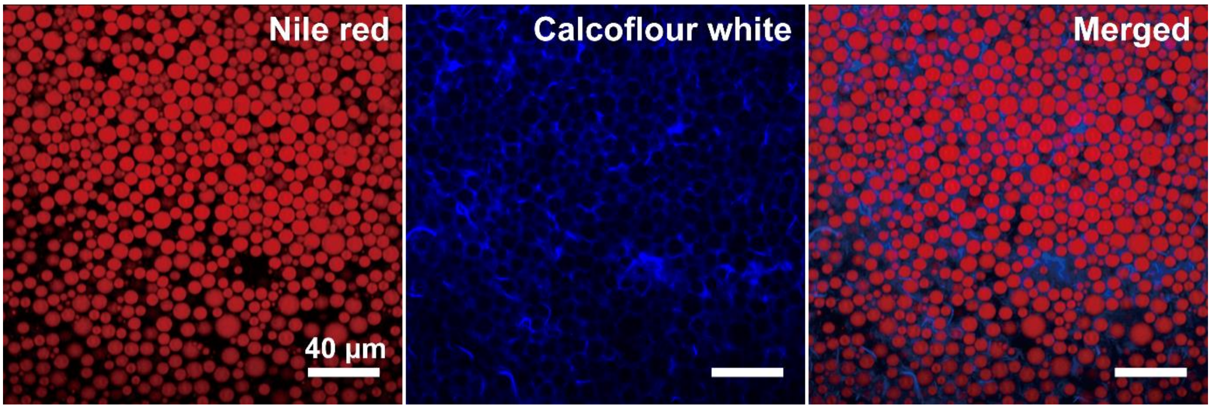

Figure S4. Confocal images of Pickering emulgels stabilized by 0.6 wt% NCh suspension. The oil volume is 50%. The left, middle, and right rows correspond to the stained oil phase (cyclohexane), continuous phase (NCh), and merged images, respectively.

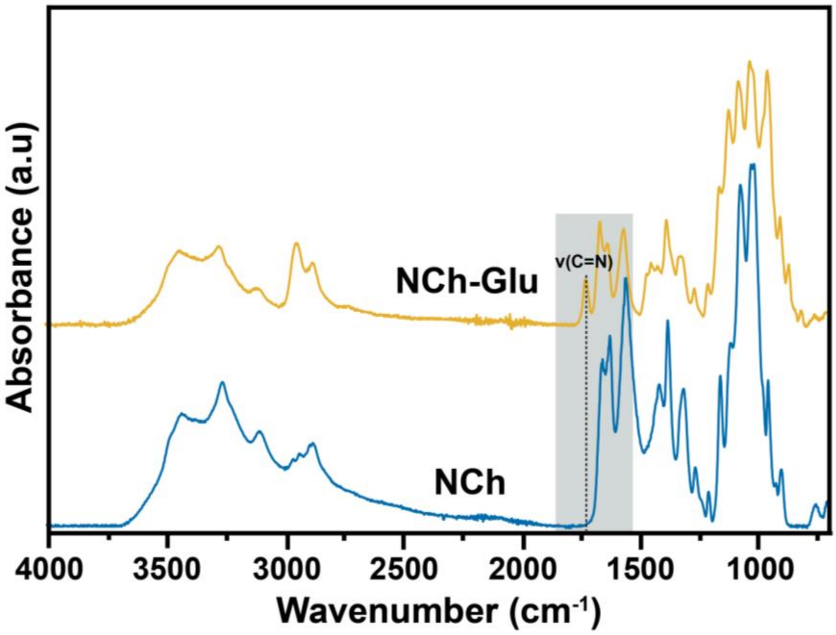

Figure S5. FTIR of NCh and NCh/Glu-0.5.

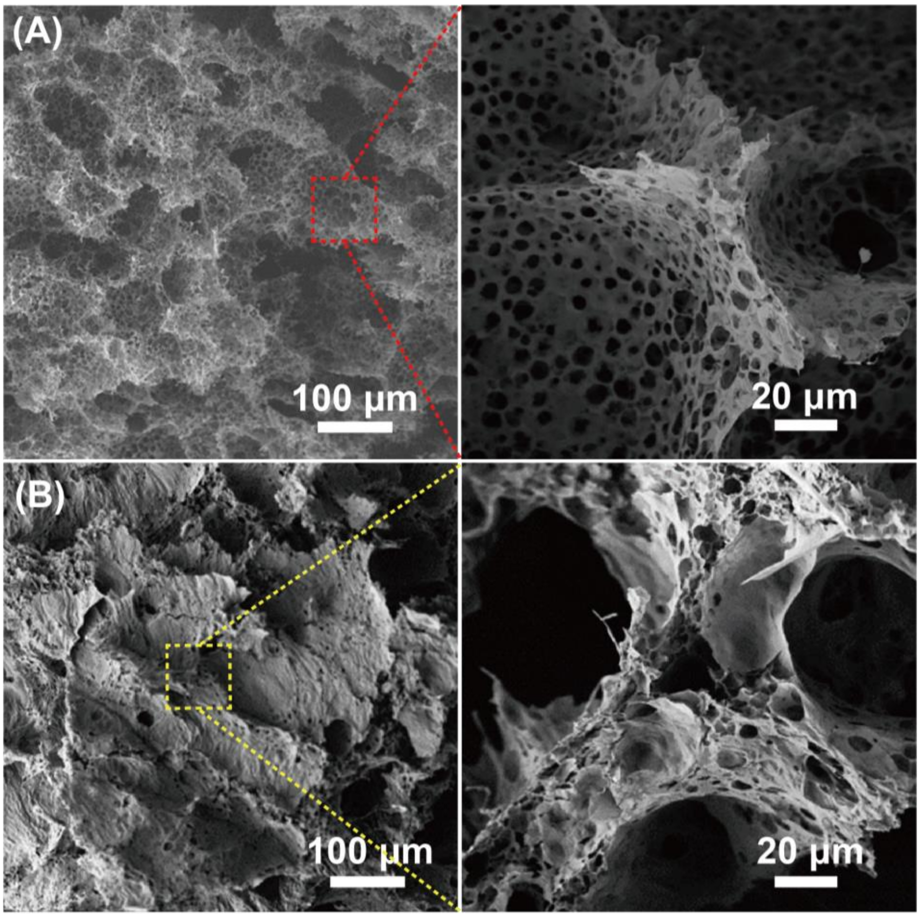

Figure S6. SEM of freeze-dried emulgels stabilized with (A) NCh/Glu-0 and (B) NCh/Glu-0.5. The red and yellow dashed lines indicate the location for magnification.

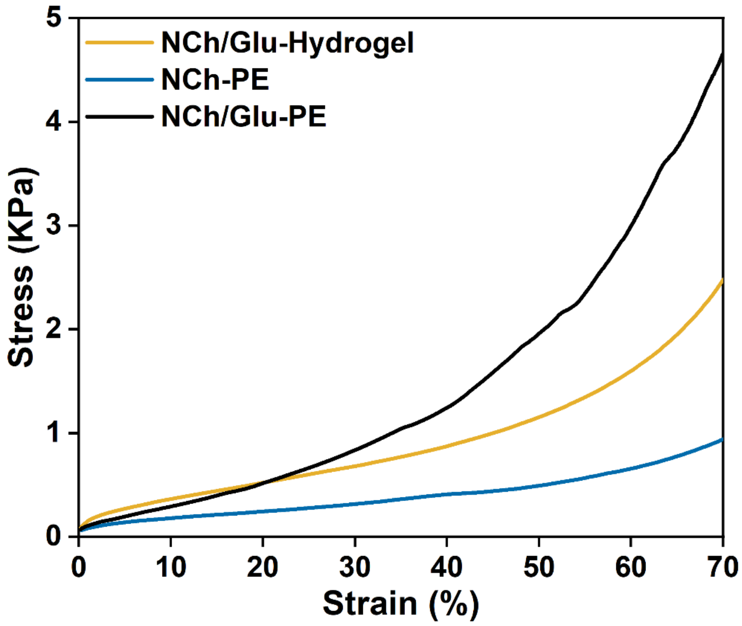

Figure S7. Compression (dynamic mechanical analysis) of the freeze-dried scaffolds prepared from different samples. The shape was the same for all the samples.

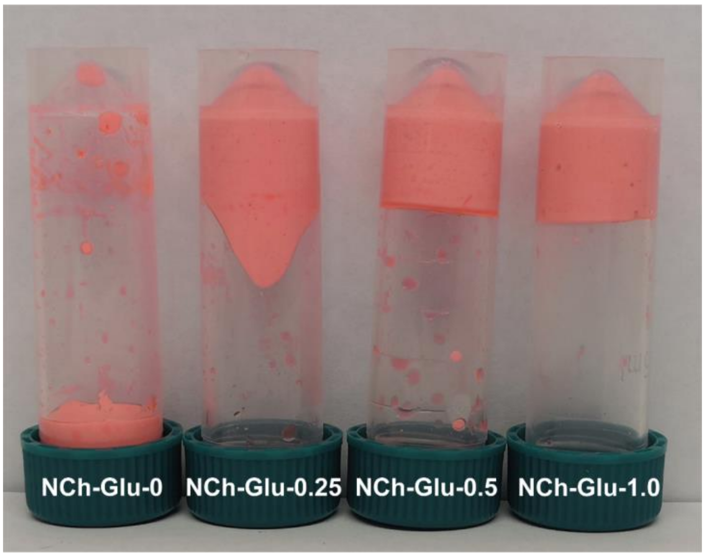

Figure S8. Visual appearance of Pickering emulgels at different NCh/Glu ratios stored in the inverted containers. Oil phase was stained by Nile red before emulsion preparation to show the oil phase were stable and homogenous in emulgels.

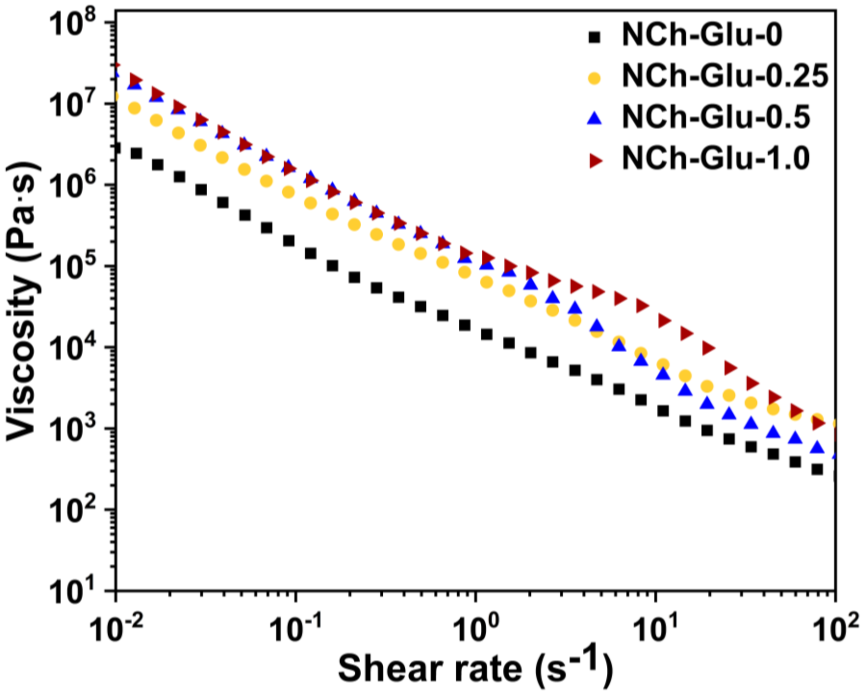

Figure S9. Shear shinning of Pickering emulgels at different NCh/Glu ratio.

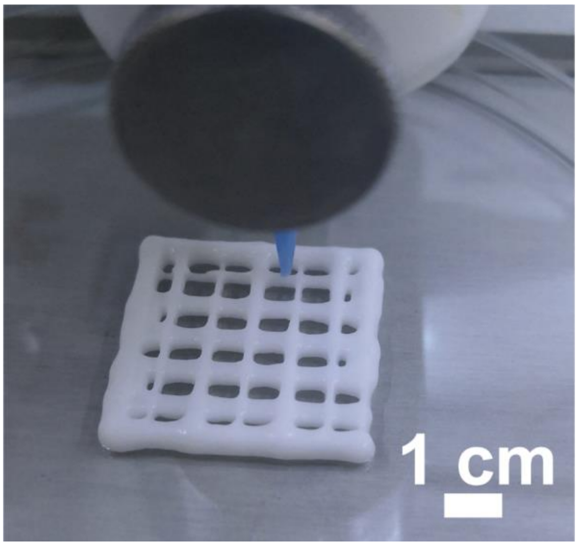

Figure S10. DIW of emulgel stabilized by NCh/Glu-0.5. The diameter of printing needle is 0.63 mm.

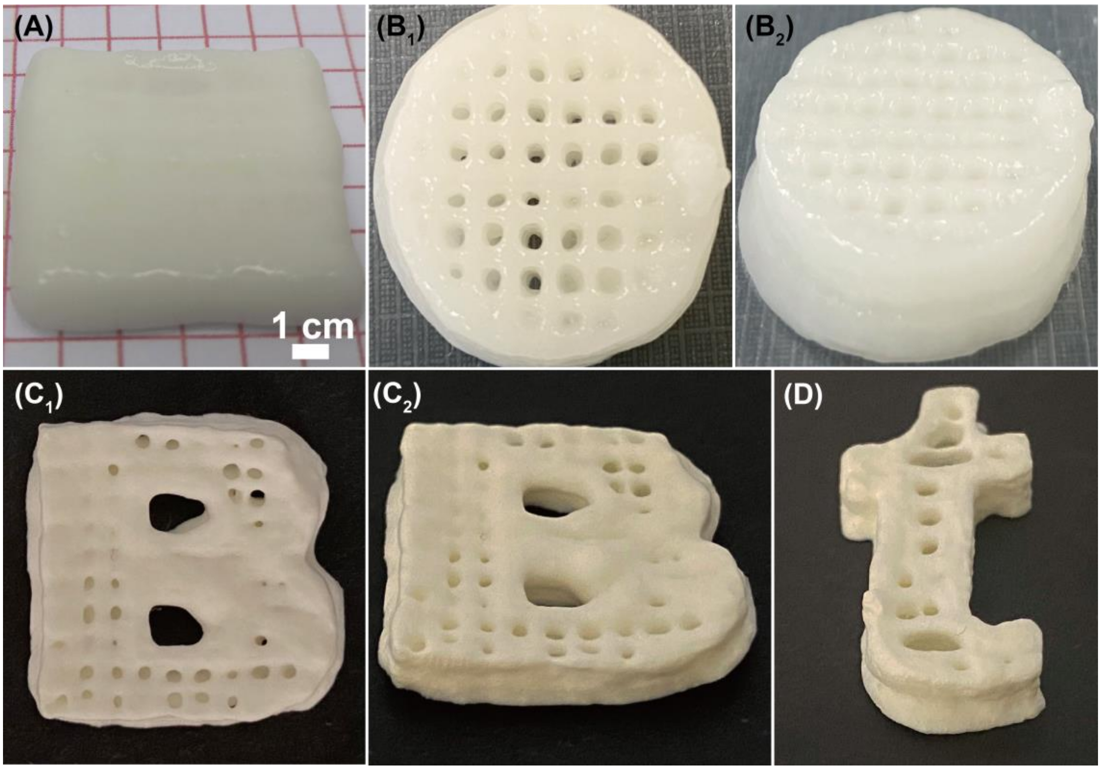

Figure S11. (A) Scaffolds printed with NCh/Glu-0.25 emulgel were unstable showing a poor infilled density. (B_1) Top and (B_2) side view of round shape scaffold (2 cm×2 cm×0.8 cm) in the wet state. The printed layers and infill density can be clearly observed upon storage. (C_1) Top and (C_2) side view of freeze-dried letter “B” that was printed using 0.25 mm needle. (D) Side view of freeze-dried letter “t”. The emulgel using in (C) and (D) was stabilized by NCh/Glu-0.5.

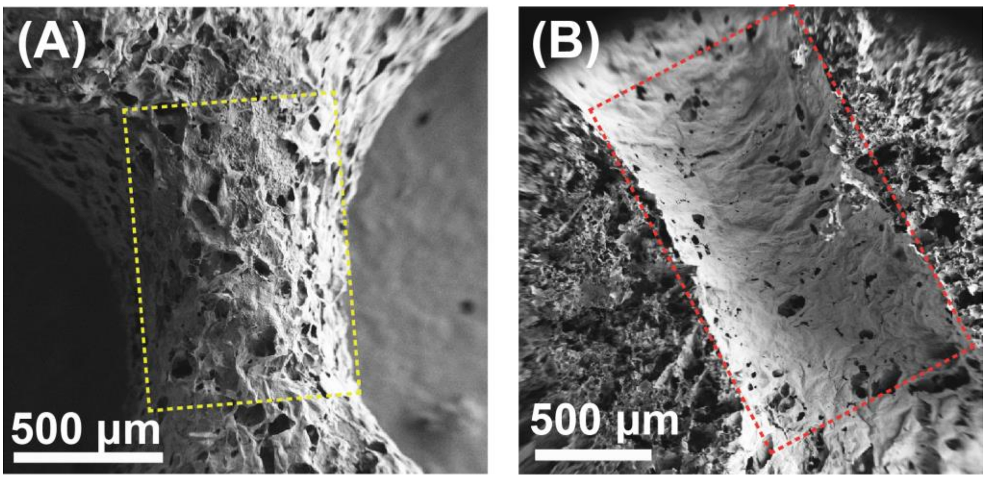

Figure S12. SEM images of (A) surface of scaffold and (B) the surface formed by squeezing a filament layer initially formed on top. The marked dashed red box indicated in (B) exhibits the same width with the filament in (A).

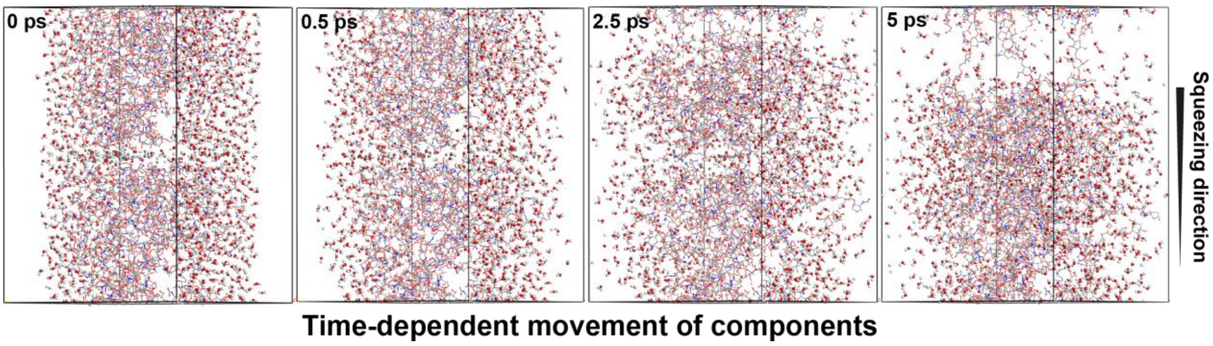

Figure S13. Snapshots of molecular dynamics (MD) simulation of relative distance between NCh and Glu molecules within 5 ps under certain pressure applied.

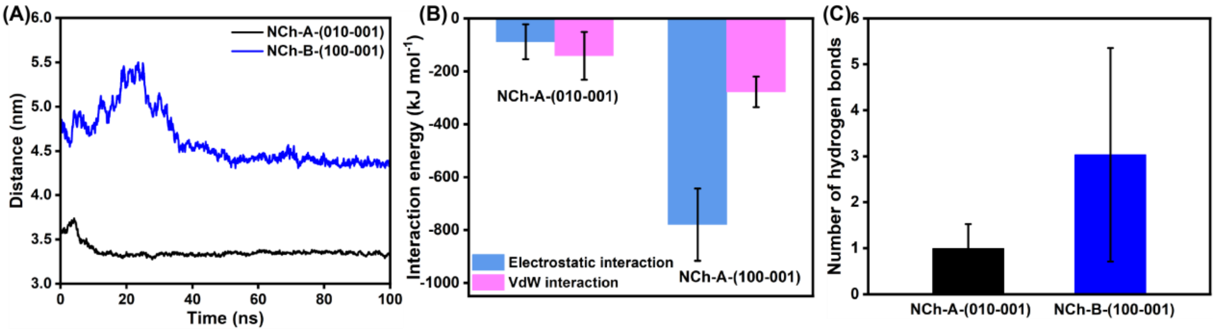

Figure S14. Classical molecular dynamics (CMD) simulation for assembly behavior of NCh, including (A) distance of center-of-mass among NChs, (B) non-bonded interactions, and (C) hydrogen bonds among NChs.

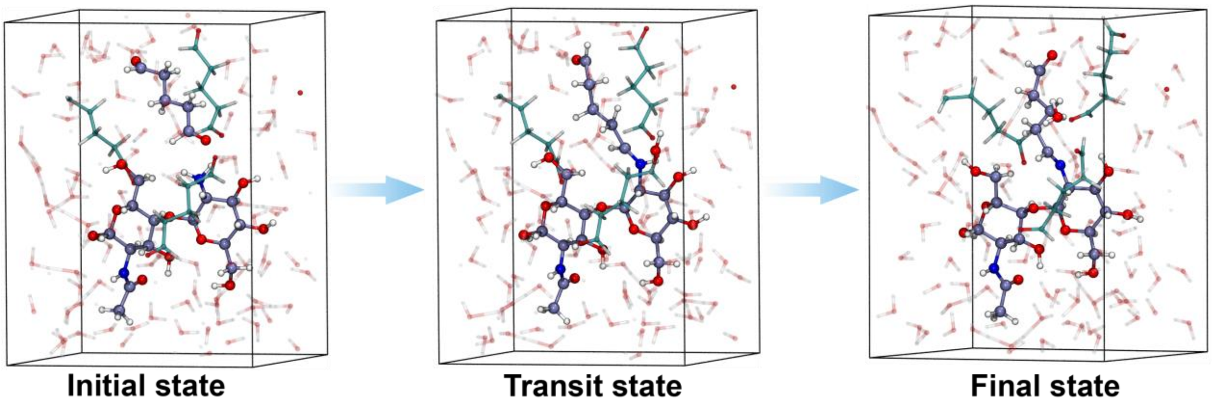

Figure S15. Snapshots of the *Ab initio* molecular dynamics (AIMD) simulation of crosslinking reaction process between NChs with Glu molecules in the Experimental section.

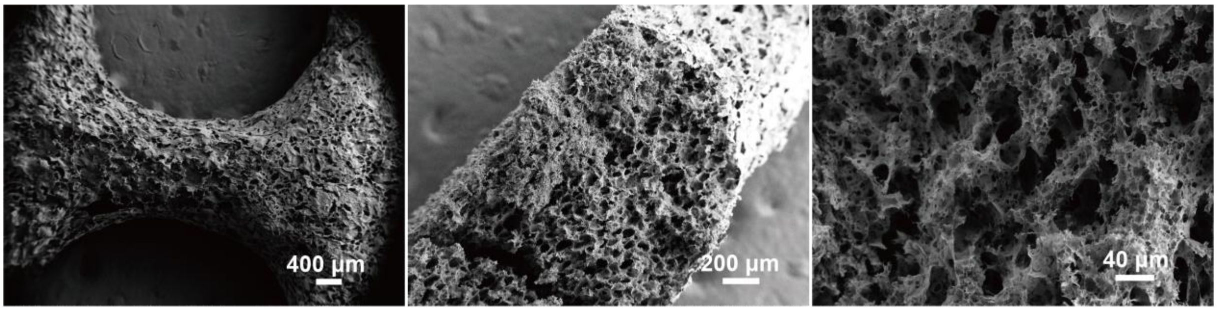

Figure S16. SEM images of surface (left panel), cross-section (middle panel) and inner (right panel) porous structure of the printed scaffold.

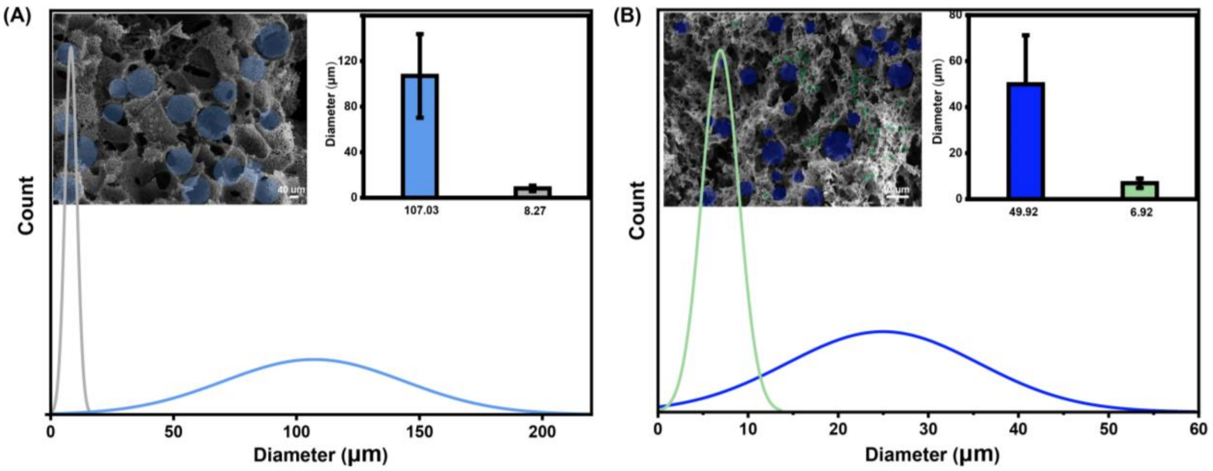

Figure S17. Average pore size of (A) NCh/Glu-PE and (B) DIW-NCh/Glu-PE. The data are calculated from the insert SEM images by ImageJ.

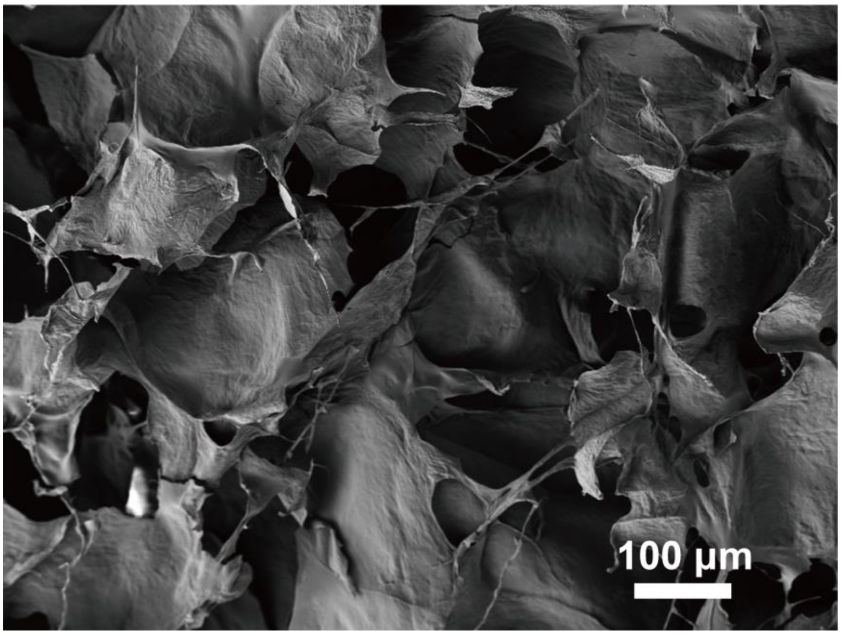

Figure S18. SEM image of the NCh/Glu-Hydrogel after freeze drying.

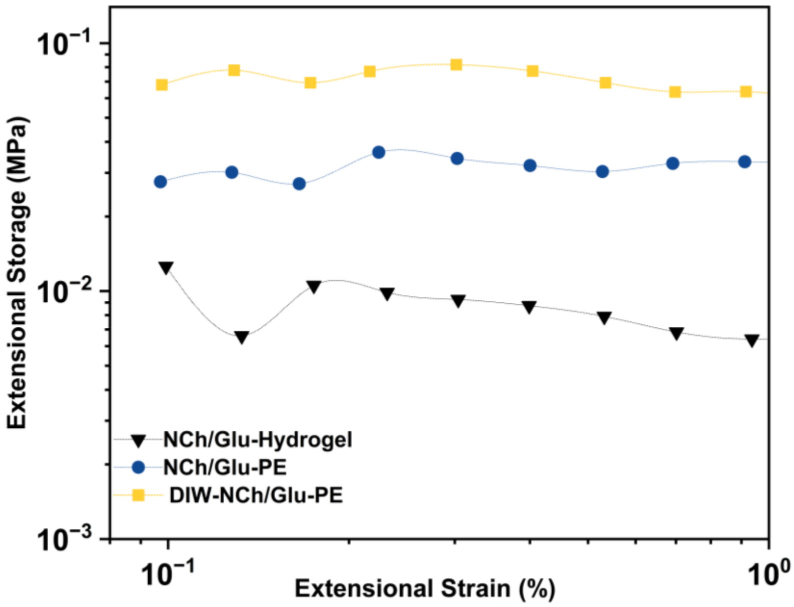

Figure S19. Extensional strain test of NCh/Glu-Hydrogel, NCh/Glu-PE and DIW-NCh/Glu-PE. All the samples were kept soaking with PBS solution during the test.

<u>Supporting table:</u>

Table S1 Composition of Pickering emulgels used for DIW printing

Sample	NCh (wt%)	Glu (wt%) [a)]	Oil (wt%) [a)]	Dry solid content (wt%)[b)]
NCh/Glu-0	0.337	0	43.8	0.337
NCh/Glu-0.25	0.333	0.277	43.2	0.609
NCh/Glu-0.5	0.328	0.547	42.6	0.875
NCh/Glu-1.0	0.319	1.064	41.5	1.383

[a)] The mass for Glu and oil to NCh was calculated according to the volume and density; [b)] Dry solid content corresponds to the mass of the printed structures after drying.

<u>Supporting videos:</u>

Video S1: Printability of NCh/Glu-0.5 Pickering Emulgel.

Video S2: 3D confocal microscopy movie of DIW-NCh/Glu-PE scaffold in phosphate buffered saline.

Video S3: 3D confocal microscopy video of MDF-GFP cells on DIW-NCh/Glu-PE Scaffolds (cell medium coated) for one day.

References

(1) Mathew, A. P.; Laborie, M.-P. G.; Oksman, K. Cross-Linked Chitosan/Chitin Crystal Nanocomposites with Improved Permeation Selectivity and PH Stability. *Biomacromolecules* **2009**, *10*(6), 1627-1632. https://doi.org/10.1021/bm9002199.

(2) Siqueira, G.; Kokkinis, D.; Libanori, R.; Hausmann, M. K.; Gladman, A. S.; Neels, A.; Tingaut, P.; Zimmermann, T.; Lewis, J. A.; Studart, A. R. Cellulose Nanocrystal Inks for 3D Printing of Textured Cellular Architectures. *Adv. Funct. Mater.* **2017**, *27*(12), 1604619. https://doi.org/10.1002/adfm.201604619.

(3) Fan, Y.; Saito, T.; Isogai, A. Chitin Nanocrystals Prepared by TEMPO-Mediated Oxidation of α-Chitin. *Biomacromolecules* **2008**, *9*(1), 192-198. https://doi.org/10.1021/bm700966g.

(4) Sun, H.; Jin, Z.; Yang, C.; Akkermans, R. L. C.; Robertson, S. H.; Spenley, N. A.; Miller, S.; Todd, S. M. COMPASS II: Extended Coverage for Polymer and Drug-like Molecule Databases. *J Mol Model* **2016**, *22*(2), 47. https://doi.org/10.1007/s00894-016-2909-0.

(5) Sikorski, P.; Hori, R.; Wada, M. Revisit of α-Chitin Crystal Structure Using High Resolution X-Ray Diffraction Data. *Biomacromolecules* **2009**, *10*(5), 1100-1105. https://doi.org/10.1021/bm801251e.

(6) Jo, S.; Kim, T.; Iyer, V. G.; Im, W. CHARMM-GUI: A Web-Based Graphical User Interface for CHARMM. *Journal of Computational Chemistry* **2008**, *29*(11), 1859-1865. https://doi.org/10.1002/jcc.20945.

(7) Martínez, L.; Andrade, R.; Birgin, E. G.; Martínez, J. M. PACKMOL: A package for building initial configurations for molecular dynamics simulations. *Journal of Computational Chemistry* **2009**, *30*(13), 2157-2164. https://doi.org/10.1002/jcc.21224.

(8) Hess, B.; Kutzner, C.; van der Spoel, D.; Lindahl, E. GROMACS 4: Algorithms for Highly Efficient, Load-Balanced, and Scalable Molecular Simulation. *J. Chem. Theory Comput.* **2008**, *4*(3), 435-447. https://doi.org/10.1021/ct700301q.

(9) Guvench, O.; Mallajosyula, S. S.; Raman, E. P.; Hatcher, E.; Vanommeslaeghe, K.; Foster, T. J.; Jamison, F. W. I.; MacKerell, A. D. Jr. CHARMM Additive All-Atom Force Field for Carbohydrate Derivatives and Its Utility in Polysaccharide and Carbohydrate–Protein Modeling. *J. Chem. Theory Comput.* **2011**, *7*(10), 3162-3180. https://doi.org/10.1021/ct200328p.

(10) Mark, P.; Nilsson, L. Structure and Dynamics of the TIP3P, SPC, and SPC/E Water Models at 298 K. *J. Phys. Chem. A* **2001**, *105*(43), 9954-9960. https://doi.org/10.1021/jp003020w.

(11) Lee, J.; Cheng, X.; Swails, J. M.; Yeom, M. S.; Eastman, P. K.; Lemkul, J. A.; Wei, S.; Buckner, J.; Jeong, J. C.; Qi, Y.; Jo, S.; Pande, V. S.; Case, D. A.; Brooks, C. L. I.; MacKerell, A. D. Jr.; Klauda, J. B.; Im, W. CHARMM-GUI Input Generator for NAMD, GROMACS, AMBER, OpenMM, and CHARMM/OpenMM Simulations Using the CHARMM36 Additive Force Field. *J. Chem. Theory Comput.* **2016**, *12*(1), 405-413. https://doi.org/10.1021/acs.jctc.5b00935.

(12) Darden, T.; York, D.; Pedersen, L. Particle Mesh Ewald: An N·log(N) Method for Ewald Sums in Large Systems. *J. Chem. Phys.* **1993**, *98*(12), 10089-10092. https://doi.org/10.1063/1.464397.

(13) Humphrey, W.; Dalke, A.; Schulten, K. VMD: Visual Molecular Dynamics. *Journal of Molecular Graphics* **1996**, *14*(1), 33-38. https://doi.org/10.1016/0263-7855(96)00018-5.

(14) Hanwell, M. D.; Curtis, D. E.; Lonie, D. C.; Vandermeersch, T.; Zurek, E.; Hutchison, G. R. Avogadro: An Advanced Semantic Chemical Editor, Visualization, and Analysis Platform. *Journal of Cheminformatics* **2012**, *4*(1), 17. https://doi.org/10.1186/1758-2946-4-17.

(15) Kühne, T. D.; Iannuzzi, M.; Del Ben, M.; Rybkin, V. V.; Seewald, P.; Stein, F.; Laino, T.; Khaliullin, R. Z.; Schütt, O.; Schiffmann, F.; Golze, D.; Wilhelm, J.; Chulkov, S.; Bani-Hashemian, M. H.; Weber, V.; Borštnik, U.; Taillefumier, M.; Jakobovits, A. S.; Lazzaro, A.; Pabst, H.; Müller, T.; Schade, R.; Guidon, M.; Andermatt, S.; Holmberg, N.; Schenter, G. K.; Hehn, A.; Bussy, A.; Belleflamme, F.; Tabacchi, G.; Glöß, A.; Lass, M.; Bethune, I.; Mundy, C. J.; Plessl, C.; Watkins, M.; VandeVondele, J.; Krack, M.; Hutter, J. CP2K: An Electronic Structure and Molecular Dynamics Software Package - Quickstep: Efficient and Accurate Electronic Structure Calculations. *J. Chem. Phys.* **2020**, *152*(19), 194103. https://doi.org/10.1063/5.0007045.

(16) Ernzerhof, M.; Scuseria, G. E. Assessment of the Perdew-Burke-Ernzerhof Exchange-Correlation Functional. *J. Chem. Phys.* **1999**, *110*(11), 5029-5036. https://doi.org/10.1063/1.478401.

(17) Schröder, H.; Creon, A.; Schwabe, T. Reformulation of the D3(Becke–Johnson) Dispersion Correction without Resorting to Higher than C6 Dispersion Coefficients. *J. Chem. Theory Comput.* **2015**, *11*(7), 3163-3170. https://doi.org/10.1021/acs.jctc.5b00400.

(18) VandeVondele, J.; Hutter, J. Gaussian Basis Sets for Accurate Calculations on Molecular Systems in Gas and Condensed Phases. *J. Chem. Phys.* **2007**, *127*(11), 114105. https://doi.org/10.1063/1.2770708.

(19) Bussi, G.; Laio, A. Using Metadynamics to Explore Complex Free-Energy Landscapes. *Nat Rev Phys* **2020**, *2*(4), 200-212. https://doi.org/10.1038/s42254-020-0153-0.

(20) Fu, H.; Chen, H.; Wang, X.; Chai, H.; Shao, X.; Cai, W.; Chipot, C. Finding an Optimal Pathway on a Multidimensional Free-Energy Landscape. *J. Chem. Inf. Model.* **2020**, *60*(11), 5366-5374. https://doi.org/10.1021/acs.jcim.0c00279.

(21) Kopolovic, I.; Ostro, J.; Tsubota, H.; Lin, Y.; Cserti-Gazdewich, C. M.; Messner, H. A.; Keir, A. K.; DenHollander, N.; Dzik, W. S.; Callum, J. A Systematic Review of Transfusion-Associated Graft-versus-Host Disease. *Blood* **2015**, *126*(3), 406-414. https://doi.org/10.1182/blood-2015-01-620872.
